# Phosphorus Oxidation Controls Epitaxial Shell Growth in InP/ZnSe Quantum Dots

**DOI:** 10.1021/acsnano.4c13110

**Published:** 2024-12-30

**Authors:** Reinout F. Ubbink, Tom Speelman, Daniel Arenas Esteban, Mourijn van Leeuwen, Maarten Stam, Sara Bals, Gilles A. De Wijs, Ernst R. H. van Eck, Arjan J. Houtepen

**Affiliations:** † Optoelectronic Materials Section, Faculty of Applied Sciences, Delft University of Technology, Van der Maasweg 9, 2629 HZ Delft, The Netherlands; ‡ EMAT Electron Microscopy for Materials Science, Department of Physics, University of Antwerp, Antwerp 2020, Belgium; § Radboud University, Institute for Molecules and Materials, Heyendaalseweg 135, 6525 AJ Nijmegen, The Netherlands

**Keywords:** quantum dots, indium phosphide, zinc selenide, photoluminescence, oxidation, interface, solid state NMR

## Abstract

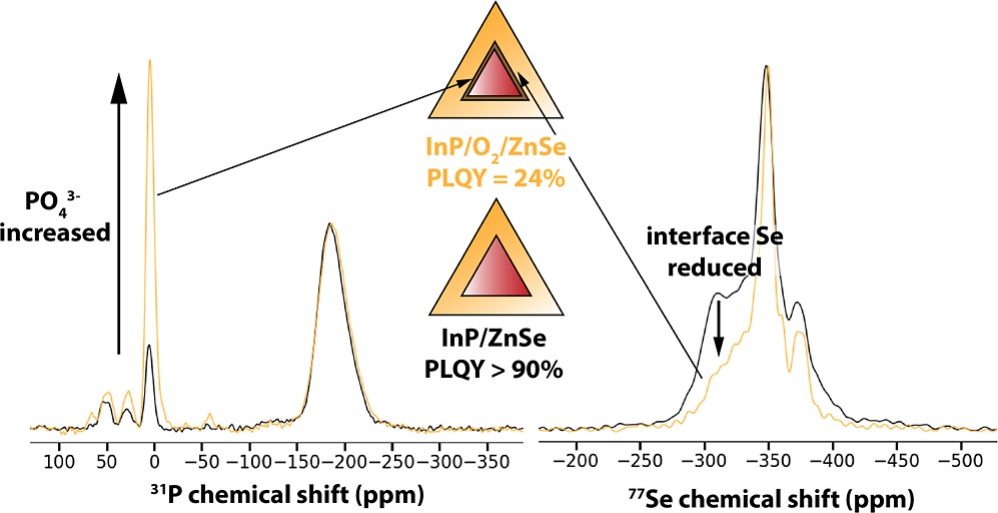

InP/ZnSe/ZnS core/shell/shell quantum dots are the most investigated quantum dot material for commercial applications involving visible light emission. The inner InP/ZnSe interface is complex since it is not charge balanced, and the InP surface is prone to oxidation. The role of oxidative defects at this interface has remained a topic of debate, with conflicting reports of both detrimental and beneficial effects on the quantum dot properties. In this study we probe the structure of the InP/ZnSe interface at the atomic level using ^31^P, ^77^Se and ^17^O ssNMR and HAADF-STEM. We observe clear differences in Se NMR spectra and crystal orientation of core and shell when the InP/ZnSe is oxidized on purpose. High levels of interface oxidation result in an amorphous phosphate layer at the interface, which inhibits epitaxial growth of the ZnSe shell.

## Introduction

Indium phosphide (InP) is the most promising ROHS-compliant material to produce quantum dots (QDs) for lighting applications.^1−3^ Although InP QDs have significantly improved over the last 5 years,^4−6^ their optical properties and stability leave much to be desired before InP QDs could be implemented as phosphors in high-intensity lighting applications such as ambient LED lamps or lasers.

One suspected source of both instability and trap states is the III–V/II–VI interface that is formed in InP/ZnSe/ZnS core/shell/shell particles. So far ZnSe and ZnS have been the most successful shelling materials for InP, leading to >90% photoluminescence quantum yields (PLQYs).^4,5,7,8^ However, ZnSe and ZnS shelling results in an inherently charged interface due to the imbalance in charges of the lattice ions.^9,10^ In addition, oxidized impurities have been identified at the InP/ZnSe interface.^4,5,11−13^ Surprisingly, the role of oxidative defects at this interface has remained a topic of debate, with reports of both detrimental^4,12,13^ and beneficial^5,11^ effects on the quantum dot properties. The nature of the exact types of oxidative defects remains ambiguous, and a description of the atomistic structure of these defects is missing.

In this work, we use solid state nuclear magnetic resonance (ssNMR) and high-angle annular dark-field scanning transmission electrons microscopy (HAADF-STEM) techniques to improve the atomistic understanding of the InP/ZnSe QD interface. The chemical nature and location of various phosphorus species on InP/ZnSe QDs are revealed through ^31^P ssNMR measurements. ^77^Se ssNMR experiments are then performed on QDs with enriched ^77^Se in the whole ZnSe shell, the InP/ZnSe interface or the ZnSe shell surface. By combining these experiments with DFT calculations, we identify selenium at different positions in the shell, distinguishing interface, bulk shell and outer surface selenium based on its chemical shift. Specifically, the ^77^Se peak at the interface is attributed to an In–Se–Zn environment at the epitaxial InP/ZnSe interface.

We then controllably oxidize the InP cores with labeled molecular ^17^O_2_ before shelling with ZnSe. This results in PO_4_^3–^ at the InP/ZnSe interface as the only reaction product. In the oxidized QDs, PLQY is significantly lower and the epitaxial In–Se–Zn ^77^Se peak, which is clearly observed in the nonoxidized QDs, is missing. This indicates that excessive oxidation disrupts the development of an epitaxial InP/ZnSe interface. HAADF-STEM measurements reveal different crystal orientations of the core and shell when the interface is oxidized, supporting the idea of a disconnected interface between the InP and ZnSe for the oxidized particles.

We propose an atomistic picture of the InP/ZnSe interface in presence and absence of oxidation. When the amount of interface oxidation is low, an epitaxial interface is grown. Excessive oxidation however disrupts the epitaxial growth, as observed by a reduction in the amount of interface selenium and the different orientation core and shell crystals. The ZnSe is separated from the InP by the amorphous oxide layer.

## Results and Discussion

### General Properties of the QDs

**Figure 1:**
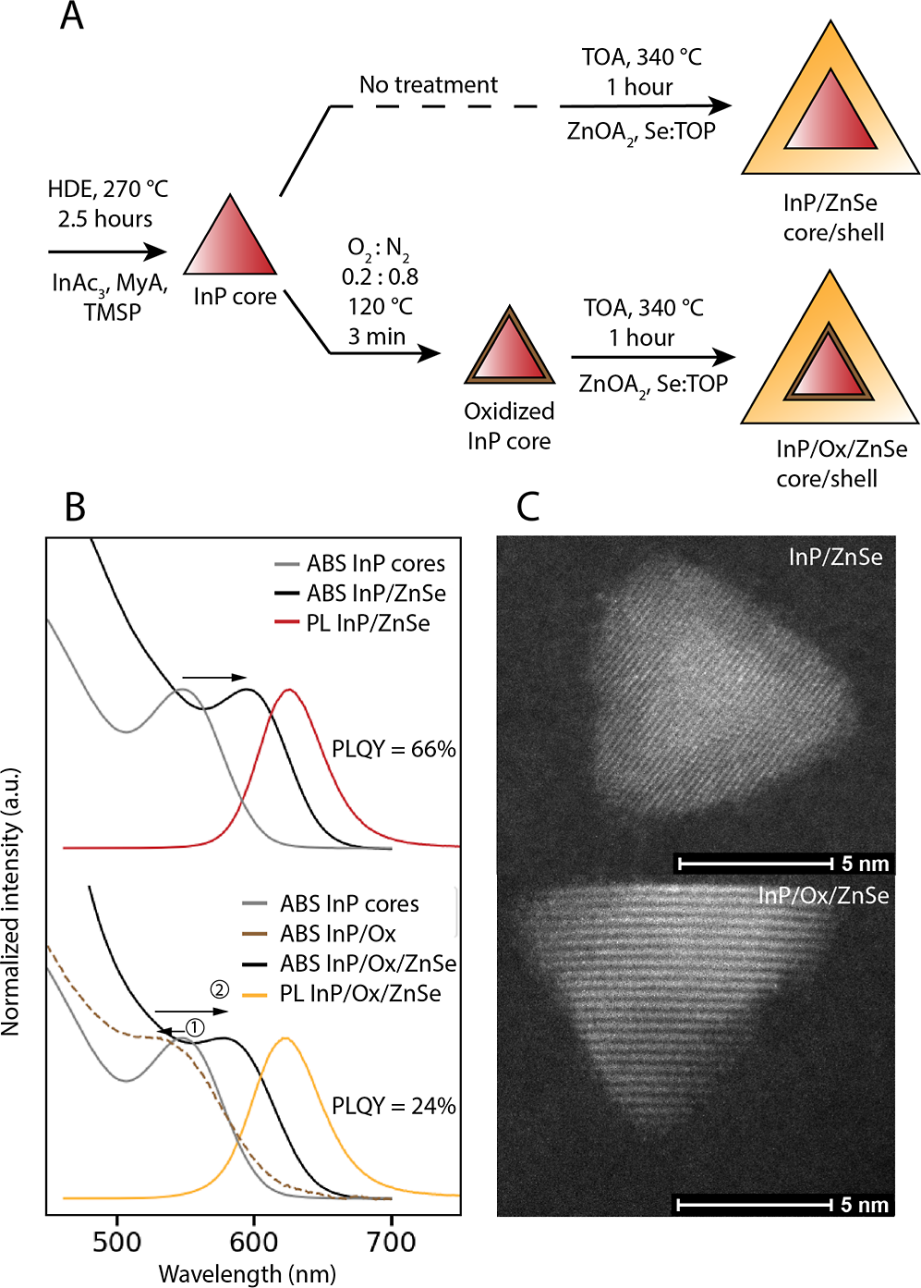
(A) The synthesis scheme of the InP/ZnSe QDs with and without an interface oxidation step. (B) Absorbance and photoluminescence spectra of the oxidized and unoxidized QDs. (C) HAADF-STEM images of the oxidized and unoxidized QDs.

Figure 1A schematically shows the protocol that was used to synthesize the InP/ZnSe QDs studied here. Zinc-free InP cores were prepared by a modified heat-up synthesis reported by Li and colleagues^7^ to minimize the surface oxidation present on the QDs.^14^ The purified cores were then heated up in the presence of zinc oleate (ZnOA_2_), and selenium dissolved in trioctylphosphine (Se:TOP) was injected dropwise for 1 h to grow the shell. As expected, the ZnSe shelling of InP particles results in a significant redshift of the absorption spectrum and an increase in the PLQY to 66% (Figure 1B). HAADF-STEM images reveal a truncated tetrahedral shape of the InP/ZnSe QDs (Figure 1C). The mean edge length of the particles increased from 3 to 6.5 nm after the shelling was completed (Figure S2). EDS analysis was performed to corroborate the core–shell atomic distribution of the QDs (Figure S3).

The synthesis method shown in Figure 1A was optimized specifically for ^77^Se ssNMR measurements. To maximize the ^77^Se signal from these samples, we did not grow an additional ZnS shell around the particles (which would “dilute” the effective density of selenium in the sample) and also applied extensive purification procedures to completely dry the samples for use in ssNMR. While this optimization results in excellent ssNMR signals from these samples, the PLQY of 66% after purification is modest compared to state-of-the-art InP core/shell/shell QDs.

To be able to generalize the results presented in this work, we also synthesized high-quality InP/ZnSe/ZnS QDs with PLQYs >90% and compared the ^31^P ssNMR results between the two methods. For this we used a similar method as shown in Figure 1A, except the shelling was performed at 280 °C in octadecene, zinc chloride was added to be able to grow a thick ZnSe shell, and an additional ZnS shell was grown. While this synthesis method resulted in high PLQY values, the ssNMR signal of these samples is too weak to accurately measure ^77^Se signal. As we will show below, there are no differences in the ^31^P measurements between these two samples, indicating the results presented here also hold for high-PLQY InP QDs. A complete comparison and analysis of the optical properties of all samples used in this study can be found in Figure S1.

To investigate the effect that oxidized interface species have on both the optical and structural properties of InP/ZnSe QDs, we introduced an oxidation step in the protocol (Figure 1A). We attempted to oxidize QDs in two ways: using either water or elemental oxygen at elevated temperatures. H_2_O oxidation was successfully performed on aminophosphine-based InP to increase interface oxidation by van Avermaet and co-workers.^5^ We attempted the same protocol, but did not see any increase in oxidation of our InP QDs in ssNMR spectra (Figure S4). We speculate that the high concentration of strongly bound apolar ligands on our QDs as compared to aminophosphine InP QDs^15^ may prevent water from reaching the QD surface before it is evaporated from the solvent.

When exposing our InP QDs to elemental O_2_ gas at elevated temperatures however we observed clear evidence of oxidation on the InP QDs, so we used isotopically enriched ^17^O_2_ gas (allowing ssNMR analysis of the oxygen) to synthesize InP/ZnSe QDs with increased oxidation at the interface. This was done by placing the QD solution under an atmosphere of 0.21 ^17^O_2_/0.79 N_2_, heating the solution to 120 °C for 3 min and then evacuating the oxygen/nitrogen mixture for 30 min (see Methods Section for details). After all oxygen had been removed, ZnSe shells were then grown following the same shelling procedure as used for the other ssNMR samples. The InP/ZnSe QDs that were obtained after treatment of the cores with O_2_ are referred to as InP/Ox/ZnSe going forward.

Both the PL and absorption of the InP/Ox/ZnSe QDs are blueshifted (absorbance maximum at 578 compared to 594) and broadened compared to the unoxidized InP/ZnSe QDs (Figure 1B). As shown below, this effect is due to the conversion of some phosphorus in the cores to PO_4_^3–^ during the oxidation, resulting in a net decrease of the InP particles size. A large difference in PLQY values is observed, as the InP/Ox/ZnSe show a PLQY of only 24%, significantly lower than the 66% that was observed for unoxidized InP/ZnSe QDs. HAADF-STEM (Figures 1C and S2) results indicate that the InP/Ox/ZnSe particles have a similar size as the unoxidized InP/ZnSe (mean edge length 7.1 nm vs. 6.5 nm). We will now first discuss the structural analysis of the unoxidized QDs and then evaluate the structural differences observed in the QDs with an oxidized interface.

### Phosphorus ssNMR

**Figure 2:**
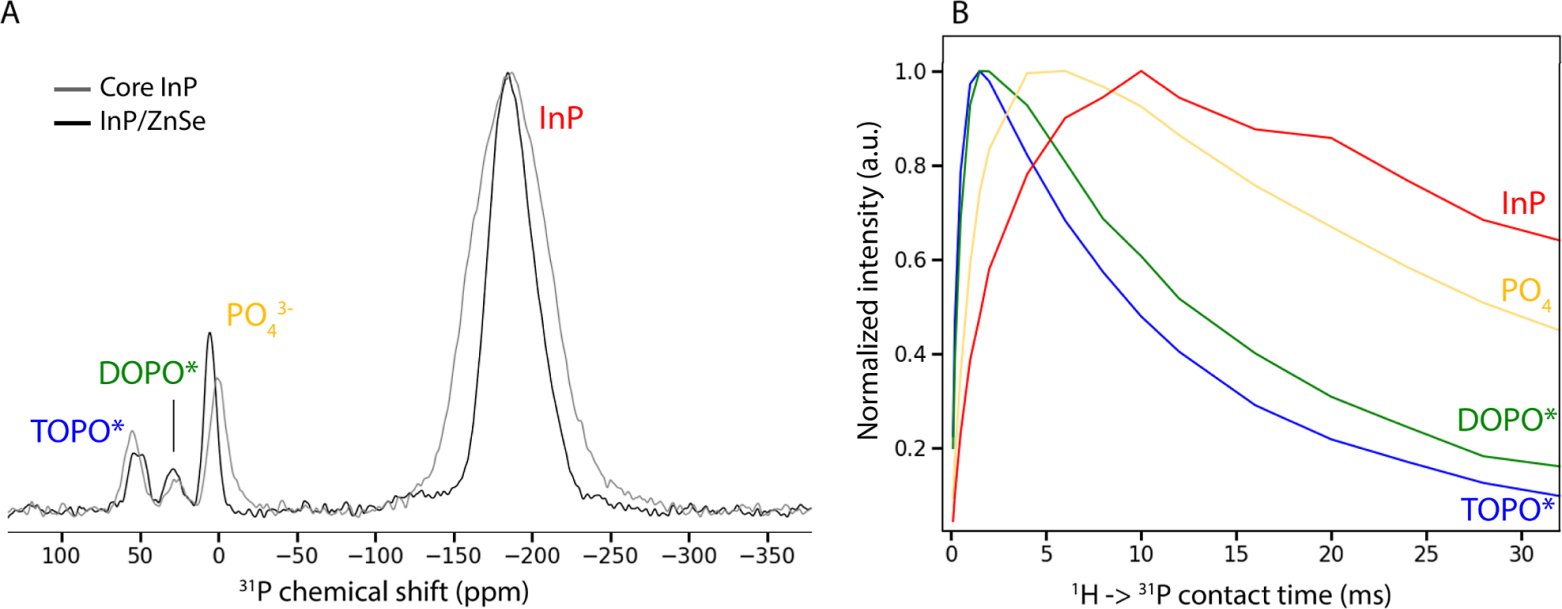
^31^P ssNMR measurements of InP QDs before and after ZnSe shelling. (A) Single pulse ^31^P measurements. The same 4 different phosphorus species are distinguished in the QDs before and after ZnSe shelling. (B) Intensity of the 4 different species in the InP/ZnSe sample for different ^1^H → ^31^P cross-polarization contact times. High intensities at short contact times indicate proximity to the hydrogen-rich ligands on the outer surface, while longer risetimes of the intensity indicate a position deeper inside the QD, removed from the surface ligands. *These peaks could also belong to other, similar organophosphorus species.

Figure 2A shows quantitative single pulse ^31^P ssNMR spectra of the InP core QDs before shelling, and of the InP/ZnSe QDs after shelling. The same 4 different species of phosphorus are distinguished in the ^31^P ssNMR spectra of the core and core/shell QDs (Figure 2A): at around −185 ppm, the typical InP peak is observed, ascribed to P^3–^ in the InP crystal.^11,16−18^ The peak around 0 ppm is ascribed to oxidized phosphorus in the form of PO_4_^3–^.^11,17,18^ We previously reported that using the heat-up synthesis it is possible to synthesize PO_4_^3–^-free InP cores,^19^ however later observations suggest that this only holds true for short synthesis times. The cores used in this synthesis were grown at 270 °C over a period of 2.5 h. As was shown previously this prolonged exposure to high temperature results in a condensation reaction of the palmitic acid precursor. The produced water is suspected to cause oxidation of surface phosphorus,^17,18^ leading to the observed PO_4_^3–^ presence on the QDs (6.9% PO_4_^3–^ out of total phosphorus). The relative integral of the PO_4_^3–^ peak is also increased somewhat after shelling, which indicates that some additional oxidation may occur in the early stages of the shelling procedure by the same precursor condensation reaction as mentioned above (percentage PO_4_^3–^ increases from 6.9% to 9.0%).^12^ Two more peaks are observed, at 28 and 50 ppm, which we ascribe to the phosphorus-containing surface ligands dioctylphosphine oxide (DOPO) and triocytlphosphine oxide (TOPO) respectively. Both these compounds are already present as contaminants in as-purchased TOP that is used in the synthesis as confirmed by solution NMR (Figure S5).^20^

^1^H → ^31^P cross-polarization experiments provide further insight into the identity and location of the 4 different phosphorus species in the core/shell QDs. In these measurements, spin polarization is transferred from hydrogen to phosphorus, increasing the ^31^P signal strength. By performing the measurement with different cross-polarization times, information can be obtained on the position of the phosphorus relative to the hydrogen in the ligands.^21^Figure 2B shows the relative signal intensity of the 4 phosphorus species after different cross-polarization (CP) times in InP/ZnSe core/shell QDs. The intensity of the InP signal rises only with longer CP times (maximum at 12 ms), since the core is separated from the ligands by the ZnSe shell and the transfer of polarization is slow. This shows that, as expected, protons are only present in ligands on the surface of the shell, and not at the interface nor in the lattice. The peaks at 28 and 50 ppm both show very strong signal intensity even at short CP times (maximum at 1.5 ms), indicating fast polarization transfer and close proximity to hydrogen. This aligns with the assignment of these peaks to DOPO and TOPO, which are bound as ligands to the surface of the QDs. During the shelling, these ligands are easily detached from the InP surface at high temperature, then reattach to the outer ZnSe surface. The PO_4_^3–^ signal reaches maximum intensity at intermediate times (6 ms) between the TOPO/DOPO and core InP peaks. This indicates that the PO_4_^3–^ is present at the interface of the InP and ZnSe, closer to the ligands than the core P^3–^ in the core, but still separated from the ligands by the ZnSe shell. This confirms earlier reports that PO_4_^3–^ species do not move to the outer ZnSe surface during the shelling procedure, but instead keep their position and are encased by the shell.^11,18^

### Selenium ssNMR

**Figure 3:**
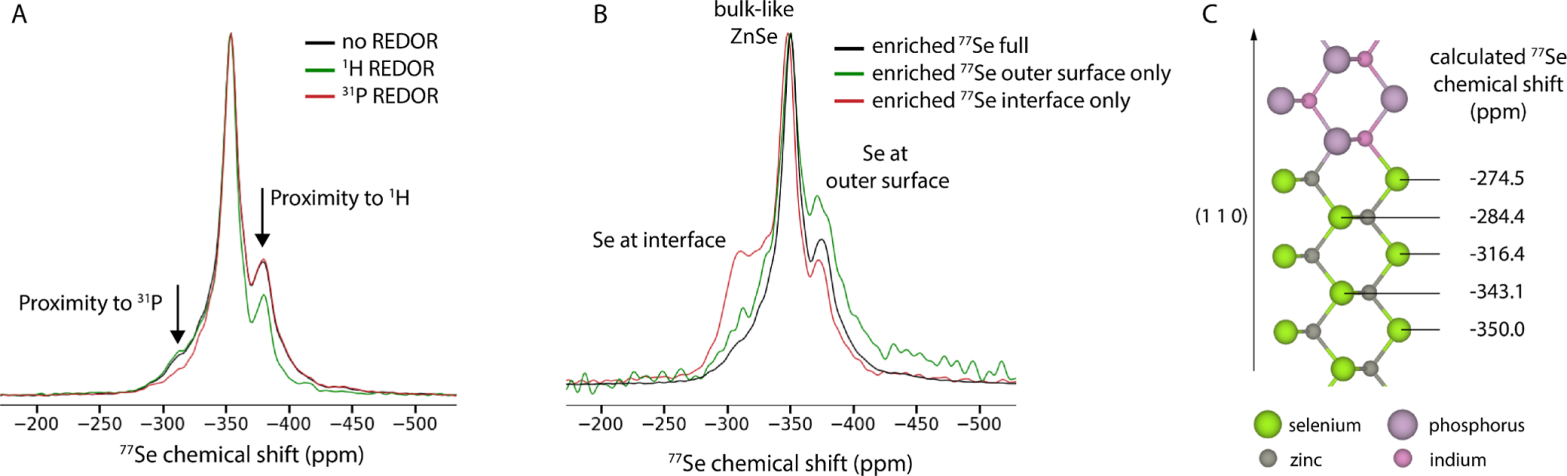
^77^Se ssNMR measurements of InP/ZnSe QDs. (A) CPMG measurements, with REDOR coupling to ^1^H and ^31^P as well as no coupling. Signals from species close to the coupled nuclei will be suppressed in the REDOR experiment. (B) CPMG measurements of QDs with enriched ^77^Se at different locations in the shell. When isotopically pure ^77^Se is placed only at the interface/outer surface of the shell, the corresponding signal at that location is strongly enhanced. (C) Calculated ^77^Se isotropic chemical shifts at the epitaxial [110] InP/ZnSe interface.

Since the natural abundance of ^77^Se is only 7.6%, InP/ZnSe QDs were synthesized with isotopically pure (>99%) elemental ^77^Se (referred to as enriched ^77^Se) to strongly enhance the ssNMR signal of the samples. Use of enriched ^77^Se increases signal from the sample by a factor 13, reducing the NMR measurement time necessary to achieve the same signal/noise ratio by a factor 13^2^ = 169. For all samples measured, ^77^Se signals were observed in the range between −250 to −500 ppm, with a maximum around −350 ppm (Figure 3A,B). This is in accordance with measurements on bulk zinc blende ZnSe.^22^ Any signals of oxidized Se species would be expected at positive chemical shifts,^23^ but were not observed, indicating that all Se in the samples was Se^2–^ in ZnSe.

Se^2–^ in different chemical environments can be distinguished by performing REDOR (Rotational Echo DOuble Resonance) measurements, correlating ^77^Se with ^1^H or ^31^P nuclei. In the REDOR sequence, the ^77^Se signal is suppressed if it comes from nuclei that are close to the correlating nucleus (either ^1^H or ^31^P). Figure 3A shows the results of both these correlation measurements compared to the ^77^Se measurement with no correlation. Selenium at more negative chemical shifts (around −380 ppm) is suppressed when correlating with ^1^H, which means that this signal corresponds to selenium atoms present at the outer surface of the shell, close to the hydrogen-rich ligands. This assignment is confirmed by ^1^H → ^77^Se cross-polarization measurements (Figure S6). In contrast, signals around −310 ppm are suppressed when correlating with ^31^P, indicating proximity to the phosphorus-rich core. We assign this peak to ^77^Se at the epitaxial InP/ZnSe interface. The main peak at −350 ppm is assigned to “bulk-like” selenium, fully coordinated by 4 zinc atoms in the zinc blende ZnSe lattice. In summary, selenium attains more negative chemical shifts the closer the selenium atoms are to the outer shell surface.

With these assignments in mind, we explored the possibility of enhancing only specific parts of the selenium signal with enriched ^77^Se. For the growth of the fully enriched ZnSe shell on our QDs, ^77^Se:TOP is injected for 60 min (see Methods Section for further details). To create interface-labeled and outer-surface labeled particles, enriched ^77^Se:TOP was instead injected for the first 5 min or last 10 min respectively, with regular Se:TOP being injected the remainder of the 60 min. The ^77^Se ssNMR measurements of these 3 different samples are compared in Figure 3B. For the interface-labeled QDs, the signal at −310 ppm is strongly enhanced, while for the outer-surface labeled QDs the signal at −380 ppm is enhanced instead. Figure 3B also shows a significantly better resolved interface peak, clearly differentiated from the more bulk-like selenium at −350 ppm. Thus, our labeling experiment confirms the previous assignments.

To help interpret the different chemical shifts observed, we performed DFT chemical shielding calculations of various selenium-based crystal structures. We reference the DFT shieldings such that for bulk ZnSe the DFT and experimental ^77^Se isotropic shift coincide, i.e. δ_calculated_ = δ_experimental_ = −350 ppm, for more details see Section S1 in the Supporting Information

We first considered the formation of a layer of mixed (non-zinc blende) In–Zn–Se crystal phase at the InP/ZnSe interface, such as recently proposed for InAs/ZnSe QDs.^24,25^ However, calculations of bulk ZnIn_2_Se_4_ and In_2_Se_3_ structures yielded shifts of −8.8 ppm and 356.1/472.3/732.3 ppm (for different selenium positions), respectively. These values are several hundreds of ppm more positive than ZnSe (at −350 ppm), which does not align with ssNMR measurements on our samples, suggesting that no separate layer of mixed In–Zn–Se crystal phase is present in our samples.

We then performed chemical shift calculations on an epitaxial (110) InP/ZnSe interface, shown in Figure 3C. The calculated ^77^Se chemical shifts of atoms next to the interface are ∼75 ppm more positive compared to selenium atoms deeper in the ZnSe material. This resembles the 40 ppm chemical shift difference between interface and bulk-like selenium atoms from our experimental data and provides additional support for the assignment of the −310 ppm peak to Se at the InP/ZnSe interface.

### Oxidation at the InP/ZnSe Interface

The presence of PO_4_^3–^ at the interface of InP/ZnSe as observed by ^31^P NMR has been reported before, with claims of both beneficial^4,12^ and detrimental^5,11^ effects on the PLQY. To prober the structural effects of an oxidized interface on the QDs, we synthesized the aforementioned InP/Ox/ZnSe QDs (Figure 1) with ^77^Se present only at the interface (injecting enriched ^77^Se for the first 5 out of 60 min of shelling).

**Figure 4:**
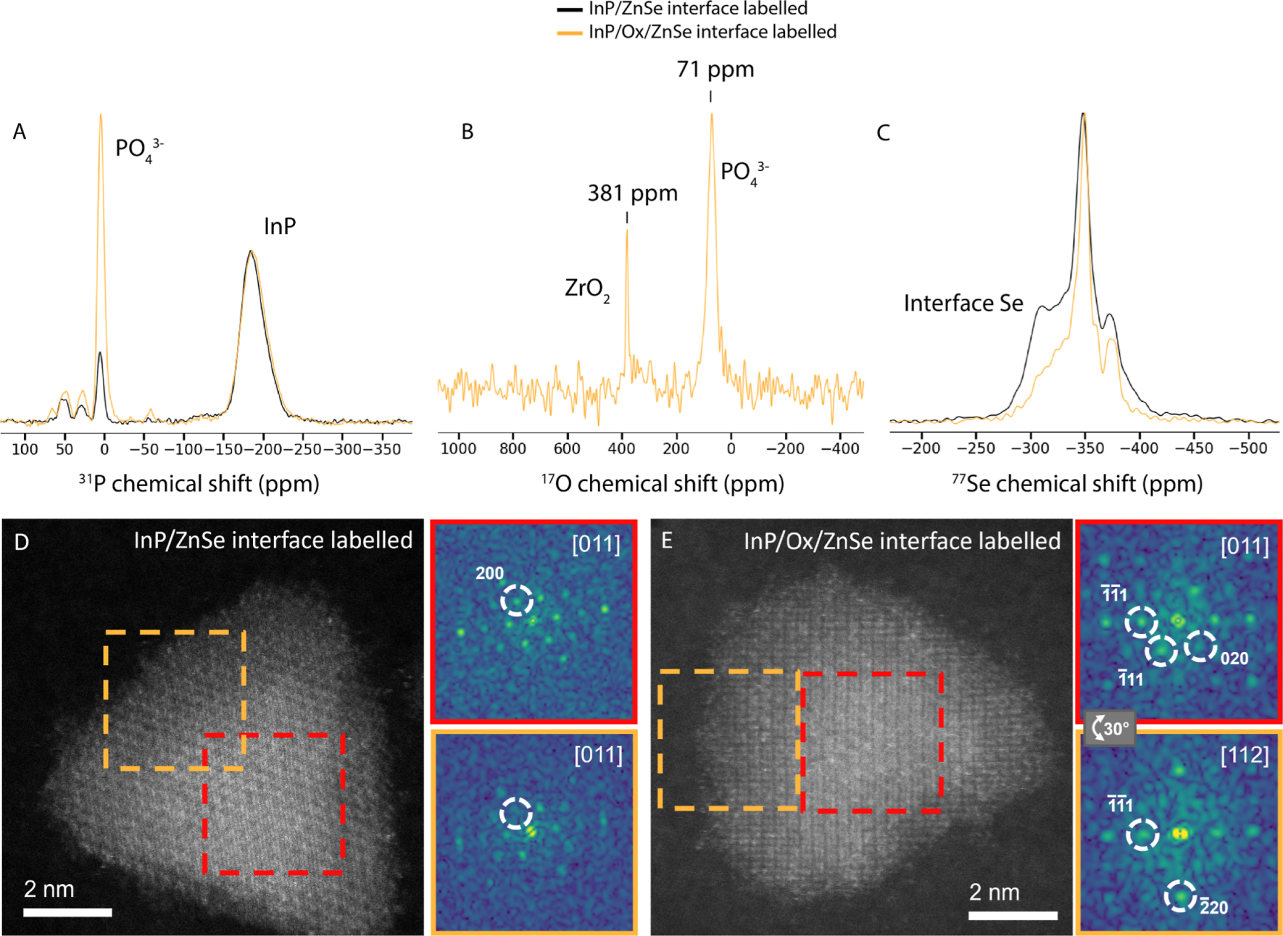
Comparison of ssNMR measurements of oxidized and nonoxidized InP/ZnSe QDs. (A) Single pulse ^31^P spectra. (B) ^17^O spectrum of the oxidized InP/Ox/ZnSe QDs. The peak at 381 ppm results from naturally abundant ^17^O from zirconia in the rotor. Only one other peak is observed from the sample in the ^17^O spectrum, assigned to PO_4_^3–^ formed during the reaction with molecular oxygen. (C) CMPG ^77^Se spectra with enriched ^77^Se at the InP/ZnSe interface. High-resolution HAADF-STEM images of nonoxidized (D) and oxidized (E) QDs. Inset shows FFT analysis of the central part (red) and external area (orange). In the nonoxidized quantum dot (QD), the crystal structures remain aligned along the [011] zone axis, displaying the characteristic fading of the (200) spacing for the ZnSe phase (white dashed circles). In contrast, the oxidized QD exhibits differing crystal orientations; the center is aligned along [011], while the outer region is aligned along [112]. These orientations are tilted at an angle of 30°, indicating the coexistence of different crystal orientations in the QD.

XPS measurements showed no difference between the oxidized and unoxidized QDs (Figure S7). Using ssNMR however, clear effects of the oxidation on the InP/ZnSe interface can be observed, as shown in Figure 4.

From the ^31^P spectra (Figure 4A), it is obvious that the amount of PO_4_^3–^ of the QDs is significantly increased. This can be quantified by dividing the integral of the PO_4_^3–^ peak by the total integral of the PO_4_^3–^ and InP peaks, yielding an increase in ^31^P in the PO_4_^3–^ state from 9.0% for the as synthesized QDs to 32.2% for the oxidized QDs. ^17^O ssNMR shows only one peak (Figure 4B), which is in the chemical shift range typically associated with phosphate species. Because there are no other peaks (except the zirconia artifact from the rotor), we conclude that all the molecular oxygen gas that reacted during our oxidation procedure ended up as phosphate species on the InP surface (which becomes the InP/ZnSe interface after shelling). In particular, we note that no In_3_O_2_, In_3_OH_3,_ nor any other hydroxyl species are observed in the ^17^O spectrum.^26^ We propose a simple reaction for the oxidation of InP with molecular oxygen at 120 °C
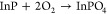
1

Selenium oxide species were again not observed. Looking at the ssNMR data of all investigated nuclei together, only one type of oxygen-containing species is actually observed at the InP/ZnSe interface: PO_4_^3–^.

### Effects of Interface Oxidation on the Properties of InP/ZnSe QDs

Earlier reports show that PO_4_^3–^ does not directly cause trap states in the InP band gap: near unity PLQYs can be obtained even when a significant amount of phosphate is present on the surface/interface of the QDs.^5,19^ When running the synthesis optimized for high PLQY (in ODE with the addition of a ZnS shell, details in the Method Section), we were also able to obtain >90% PLQY on the same InP cores. While these QDs were not intentionally oxidized, they did show a significant presence of PO_4_^3–^, even after shelling (Figure S4). This PO_4_^3–^ was again confirmed to be situated at the InP/ZnSe interface by ^1^H → ^31^P cross-polarization experiments (Figure S4), as explained in Figure 2B. Through integration of ssNMR peaks and atom counting^27^ it can be estimated that each QD, even when not intentionally oxidized, still contains an average of 5 PO_4_^3–^ moieties. Since this sample still shows a PLQY of >90%, this demonstrates that the mere presence of interface PO_4_^3–^ is not enough to cause carrier trapping and recombination. This is in line with our earlier DFT results that showed no addition of states in the bandgap when PO_4_^3–^ is present on the surface. Rather, new orbitals associated with PO_4_^3–^ reside inside the valence band.^19^

However, the presence of phosphate species on the interface could still lead to in-gap states indirectly, by increasing disorder at the interface, which may result in e.g. undercoordinated interface atoms. Figure 4C shows the ^77^Se spectra of the oxidized and unoxidized QDs. Both samples contain enriched ^77^Se spectra only at the interface, strongly enhancing the signal of selenium nuclei that are closest the InP/ZnSe interface.

In the oxidized sample, a clear peak at −310 ppm is missing, even though the same amount of enriched ^77^Se is present in this sample. Instead, only bulk-like ^77^Se is present at −350 ppm, as well as Se at the outer ZnSe shell surface at −380 ppm. This indicates that the PO_4_^3–^ layer interrupts the crystal at the InP/ZnSe interface, resulting in a decreased amount of selenium at epitaxial interface positions.

This interruption of the crystal is also supported by HAADF-STEM measurements of non-oxidized and oxidized QDs. Both InP and ZnSe have the same zinc blende cubic crystal structure with space group *F*4̅3*m*. However, a noticeable decrease in the intensity of the (200) crystal plane in the ZnSe system compared to InP makes it possible to distinguish between the crystal structures present in the same nanoparticle.^28^ This difference is shown clearly in a HAADF-STEM image of a QD oriented along the [001] crystal direction in Figure S2 in the Supporting Information.

In Figure 4D, a nonoxidized QD is shown, with the crystal structure from the center and border area remaining oriented along the same [011] direction. When performing a fast Fourier transform (FFT) on the shell region (orange square), only a faint (200) crystal spacing is observed, highlighted by a white dashed circle in the FFT analysis, characteristic for the ZnSe phase. When performing the same analysis in the center of the particle (red square), the (200) spacing is much more clearly visible, indicating that InP is present in addition to the ZnSe shell in this part of the particle. The alignment of both crystal structures along the same zone axis indicates that the core and shell crystals have the same structure and orientation, pointing to epitaxial growth of the ZnSe on the InP core. In contrast, Figure 4E shows an oxidized QD, where the crystalline structure from the center of the particle exhibits a different crystal orientation of [011] compared with the shell crystal structure of [112]. This suggests a tilt of 30° between the two structures. The center of the QD additionally shows a blurred effect most likely due to the oxide layer created between the core and the shell. This observation indicates that the shell was grown in a different orientation than the core InP. The excessive presence of PO_4_^3–^ in the oxidized InP/ZnSe thus appears to disrupt the zinc blende lattice, interfering with epitaxial shell growth.

While we showed that excessive interface oxidation is detrimental to the PLQY of our InP/ZnSe QDs, we were also able to obtain near-unity PLQY values with significant presence of interface phosphate (an average of 5 PO_4_^3–^ moieties per QD, Figures S1 and S4). Although it is difficult to imagine how these are incorporated in the zinc blende crystal at the InP/ZnSe interface, apparently their presence does not result in trap states. The disruption of epitaxy discussed above only seems to occur when even more interface oxidation occurs.

**Figure 5:**
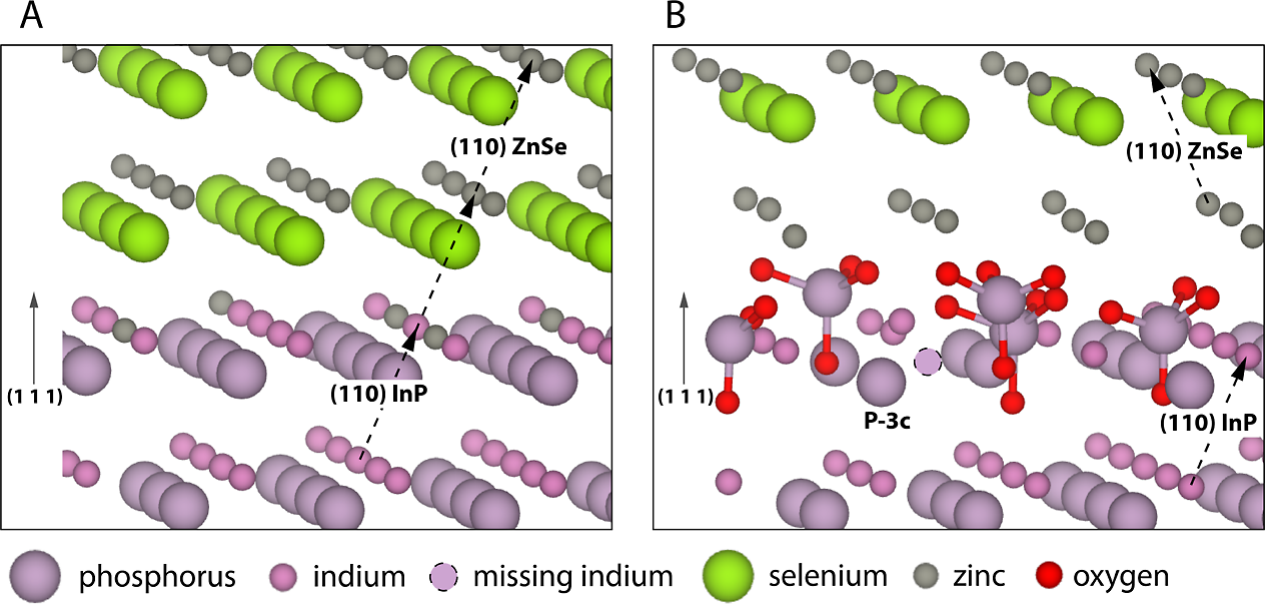
Sketches of the proposed structure of the InP/ZnSe interface with and without oxidation. (A) Oxide-free InP/ZnSe (111) interface. The proposed oxide-free interface is an epitaxial InP/ZnSe interface, where the ZnSe crystal is oriented in the same direction as the InP crystal (dashed arrows). Selenium is present at the epitaxial interface, resulting in the characteristic NMR resonance observed around −310 ppm. (B) Oxidized InP/ZnSe (111) interface. Most of the P^3–^ has been converted to PO_4_^3–^, preventing the binding of selenium at the interface until a layer of positive zinc ions is present. The PO_4_^3–^ layer disrupts the crystal lattice, which may result in trap states, for example an undercoordinated phosphorus (shown in B as *P*3̅*c*) and growth of the ZnSe crystal in a different orientation (shown by dashed arrows) as observed in HAADF-STEM images. Bonds between phosphorus and oxygen are shown in B to highlight the phosphate units. Other bonds were omitted for visual clarity.

Figure 5 shows schematically the effects that excessive oxidation can have on the interface based on the results of this work. In this schematic, an amorphous phosphate layer is formed on top of the InP particles, in correspondence with the ssNMR observations. When shelling on the oxidized particles is performed, selenium ions cannot directly bind to indium. Instead, zinc binds first to the phosphate, and only then can selenium bind to begin the formation of the ZnSe lattice. The signal at −310 ppm that is observed from selenium at the epitaxial InP/ZnSe interface is thus suppressed.

## Conclusion

This study elucidates the atomic structure of the InP/ZnSe QD interface with and without oxidation. It is shown how a high level of oxidation results in the presence of an amorphous phosphate layer at the InP/ZnSe quantum dot interface, which inhibits epitaxial shell growth. At the same time, it is shown that fully oxide-free interfaces are not required to obtain high-quality InP QDs, as epitaxial growth is still possible even when some phosphate is present. By providing insight in the effects of interface oxidation, these findings will aid the development of stable and high-quality QDs for lighting applications.

## Methods

### Materials

Anhydrous indium acetate (Thermo Fisher, 99.99%), anhydrous zinc acetate (Thermo Fisher, 99.9%), anhydrous zinc chloride (Merck Sigma, 99.999%), myristic acid (Merck Sigma, >99%), tris(trimethylilyl)phosphine (TMSP, Strem, >98% *Caution: TMSP is a highly pyrophoric substance that can release toxic phosphine gas upon reaction with air*), selenium (Merck Sigma, 99.99%), selenium-77 (CortecNet, enrichment level 99.66%), sulfur (Alpha Aesar, 99.9995%), anhydrous acetone (VWR, max 0.01% H_2_O), anhydrous toluene (VWR, 20 ppm of H_2_O) and anhydrous ethanol (VWR, 30 ppm of H_2_O) were used as received. Oleic acid (Merck Sigma, 90%), octadecene (ODE, Merck Sigma, 90%), trioctylamine (TOA, Merck Sigma >92.5%) and trioctylphosphine (TOP, Merck Sigma, 97%) were degassed in vacuo at 120 °C for at least 1 h before use. All chemicals were stored inside a glovebox under inert N_2_ atmosphere (concentration O_2_ and H_2_O < 0.1 ppm).

### InP Core QD Synthesis

InP cores were synthesized according to a protocol by Li et al. 450 mg indium acetate (1.54 mmol), 1056 mg myristic acid (4.62 mmol) and 55 mL hexadecane were combined in a 3-neck roundbottomflask inside a nitrogen-filled glovebox (concentration O_2_ and H_2_O < 0.1 ppm). The flask was closed, taken outside and attached to a Schlenk line, were the mixture was degassed in vacuo for 30 min at room temperature under constant stirring. After purging, the flask was put under an atmosphere of Ar/2%H_2_ and a needle was then added to bubble Ar/2%H_2_ gas through the reaction mixture for the rest of the procedure (flow rate ∼0.2 L/min). The mixture was raised to 150 °C and kept at that temperature for 30 min, during which the myristic acid and indium acetate reacted to form indium myristate and acetic acid, which was carried away by the bubbled gas. Then, 6750 mg of trioctylphosphine (TOP) was injected into the mixture. Once the temperature recovered to 150 °C, 9000 mg of 0.063 M tris(trimethylsilyl)phosphine (TMSP) in hexadecane was injected into the mixture as quickly as possible, using two syringes (*Caution: TMSP is a highly pyrophoric substance that can release toxic phosphine gas upon reaction with air*). This led to an instant color change of the mixture from colorless to yellow as the InP nucleation took place. The temperature was raised to 270 °C. After 10 min, an additional 15 mL of 0.010 M TMSP in hexadecane was injected over 2.5 h to grow the InP crystals. After cooling down, this yielded a dark red InP QD solution, which was returned under Ar/2%H_2_ atmosphere to the glovebox. This crude mixture was purified by addition of 4 volume equivalents of anhydrous acetone, followed by centrifugation at 6000 rpm for 10 min. The clear supernatant was discarded, leaving a liquid residue which was redissolved in 24 mL of anhydrous toluene. Another 4 volume equivalents of anhydrous acetone was added, and after centrifugation at 6000 rpm for 40 min and discarding supernatant, the solid residue was redissolved in anhydrous toluene, yielding the InP core QD solution.

### Treatment of InP Cores with H_2_O

126.5 μL of InP core solution (0.033 μmol InP QDs) was mixed with 3 mL of octadecene in a three-neck roundbottomflask inside a glovebox. The flask was taken outside, connected to a Schlenk line and degassed in vacuo for 10 min. The temperature was raised to 120 °C, and a drop of water (11 mg = 0.61 mmol, ratio indium atoms/water molecules = 65) was injected using a syringe. The mixture was stirred at 120 °C for 10 min under Argon bubbling, after which it was evacuated for 60 min at 110 °C to remove any excess water. The QD solution was purified three times by addition of 4 equiv of anhydrous acetone, centrifugation at 6000 rpm for 10 min and redispersion in anhydrous heptane.

### Preparation of ZnOA_2_

Zinc oleate precursor in octadecene (ZnOA_2_ in ODE, 0.46 M) was synthesized by mixing 3.38 g zinc acetate (18.4 mmol), 10.4 g oleic acid (36.8 mmol) and 40 mL of ODE in a three-neck flask inside a nitrogen filled glovebox. The resulting solution was transferred to a Schlenk line, degassed for 10 min, then heated to 150 °C and reacted for 60 min in vacuo until a clear solution was obtained. The solution was then transferred to a vial and stored in a nitrogen-filled glovebox.

### ZnSe Shelling of InP QDs

In a three-neck roundbottomflask in the glovebox, 957 μL of InP core solution (0.87 μmol InP QDs) was mixed with 2580 mg of ZnOA_2_ solution (0.46 M in ODE, heated to 150 °C, total ZnOA_2_ ∼ 1.8 mmol) and 80 mL trioctylamine (TOA). The flask was sealed, taken outside and attached to a Schlenk line. The mixture was degassed in vacuo for 30 min at room temperature and 30 min at 120 °C under constant stirring. If the oxidation was performed, the atmosphere was changed to 80%/20% N_2_/isotopically enriched ^17^O_2_ before heating (*Caution: large hydrocarbons have low autoignition temperatures and care should be taken when heating these solvents in an oxygen-rich atmosphere*). The temperature was then raised to 120 °C over 18 min. Aliquots were regularly taken to check changes in the absorption spectrum of the QDs. A strong broadening was observed after 3 min at 120 °C, after which the mixture was cooled down and degassed in vacuo for another 30 min. After changing the atmosphere to pure Ar gas, the temperature was raised to 340 °C. Starting after 5 min, Se:TOP was injected into the mixture for 60 min using a syringe pump, during which the ZnSe shell growth took place. In total, 587 mg of 2.0 M Se in TOP solution was injected, diluted to 10 mL total volume in TOA (injection rate = 20.4 μmol selenium/min). Isotopically pure (enriched) ^77^Se was injected for either the first 5 min, final 10 min or full 60 min and normal elemental selenium was used for the remaining duration. The mixture was cooled down and returned to the glovebox under inert atmosphere. Purification was performed by adding 3 volume equivalents of anhydrous ethanol, centrifugation at 6000 rpm for 10 min, discarding the clear supernatant and redissolving in toluene. This process was then repeated once. A very dry, deep red powder was obtained after 2 rounds of purification and drying under vacuum.

### PLQY-Optimized InP/ZnSe/ZnS Synthesis

In a three-neck roundbottomflask in the glovebox, 360 μL of InP core solution (0.33 μmol InP QDs) was mixed with 1440 mg of ZnOA_2_ solution (0.46 M in octadecene, heated to 150 °C, total ZnOA_2_ ∼ 1.0 mmol), 60 mg ZnCl_2_ (0.44 mmol) and 48 mL octadecene (ODE). In our experience, ZnCl_2_ could not be used in conjunction with TOA, as this will cause the InP cores to accumulate and crash out of the solution. The flask was sealed, taken outside and attached to a Schlenk line. The mixture was degassed in vacuo for 30 min at room temperature and 30 min at 120 °C under constant stirring. After changing the atmosphere to pure Ar gas, the temperature was raised to 280 °C. Starting when the temperature reached 230 °C, Se:TOP was injected into the mixture for 60 min using a syringe pump, during which the ZnSe shell growth took place. In total, 220 mg of 2.0 M Se in TOP solution was injected, diluted to 5 mL total volume in ODE (injection rate = 7.6 μmol selenium/min). The solution was kept at 280 °C for 15 min, then S:TOP was injected over 30 min using a syringe pump. In total, 110 mg of 2.0 M S in TOP solution was injected, diluted to 2.5 mL total volume in ODE (injection rate = 7.6 μmol sulfur/min). The mixture was cooled down, and purified 3 times using the same method as described above for the InP/ZnSe QDs. Despite extensive purification, a liquid residue was obtained, which was dried in vacuo to obtain a sticky red paste.

### Optical Characterization

UV–vis absorption spectra were recorded on a PerkinElmer Lambda 365 spectrometer. Emission measurements were recorded on Edinburgh Instruments FLS980 spectrometer equipped with a PMT 750 detector. Photoluminescence quantum yields were measured against a fluorescein dye solution in 0.1 M NaOH in H_2_O at an excitation wavelength of 465 nm. To calculate the quantum yield, the literature value of 92% is considered for the quantum yield of fluorescein.^29^

### Transmission Electron Microscopy

High-angle annular dark field scanning transmission electron microscopy (HAADF-STEM) and energy-dispersive X-ray spectroscopy (EDS) were performed using an aberration-corrected Thermo Fisher Titan G2 60-300 electron microscope equipped with a SuperX EDS detector operated at 300 keV. For the EDS analysis, 342 frames with a pixel size of 50.75 pm were acquired using a dwell time of 2 μs and a beam current of 150 pA, resulting in a total dosage of approximately 2.5 × 10^6^ e^–^ Å^–2^ to ensure accurate element detection. X-ray diffraction relative intensity data was obtained from the Materials Project for InP (mp-20351) and ZnSe (mp-1190) from database version v2023.11.1.^28^

### X-Ray Diffraction

Powder X-ray diffraction samples were prepared by drop casting QD solutions onto low-reflection silicon wafers. XRD patterns were then collected on a Bruker D8 Advance diffractometer (Cu Kα, λ = 1.5418 Å).

### Solid-State NMR

ssNMR analysis was performed at the Magnetic Resonance Research Center at the Radboud University Nijmegen. Samples were loaded into 4 mm zirconia rotors inside a nitrogen filled glovebox. Measurements not involving ^77^Se or ^17^O were performed using an Agilent 400 MHz magnet operating at ^1^H and ^31^P resonance frequencies of 399.9 and 161.9 MHz respectively, with a CMX 4.0 mm T3 SPC400-550 probe, spinning at a MAS frequency of 10 kHz. Spectra were referenced to external H_3_PO_4_ in H_2_O (=0 ppm). Single Pulse 31P measurements were collected with a recycle delay (d1) of 600 s and a ^31^P π/2 6 μs pulse width. Extremely long recycle delays are needed to obtain quantitative data since relaxation is very slow in these highly crystalline samples with core phosphorus separated from the surface by the ZnSe shell. ^1^H → ^31^P CPMAS measurements were performed with a ^1^H π/2 pulse width of 6 μs and a recycle delay (d1) of 4 s. Proton decoupling was performed during the CP measurements using the Spinal-64 decoupling sequence. Measurements involving ^77^Se or ^17^O were performed using a Bruker 850 MHz magnet operating at ^77^Se, ^1^H, ^31^P and ^17^O resonance frequencies of 162.1, 849.7, 344.0, and 115.2 MHz respectively with an Agilent T3 4.0 mm probe, spinning at a MAS frequency of 10 kHz. All spectra were referenced to external H_3_PO_4_ in H_2_O (=0 ppm in the ^31^P). ^77^Se spectra were accumulated using a CPMG (Carr–Purcell–Meiboom–Gill) pulse sequence (see Figure S8 for the full pulse program), with a ^77^Se π/2 pulse width of 6 μs and a recycle delay (d1) of 300 s. 64 CPMG cycles were recorded with each cycle consisting of 16 rotor periods. In the REDOR measurements, either ^1^H, ^31^P or no dephasing pulses were given at half and full rotor periods, while refocusing pulses were given on the selenium channel every 16 rotor periods. The REDOR dephasing was performed for 16 ms before the CPMG signal acquisition. ^17^O spectra were accumulated using a CPMG pulse sequence with a ^17^O π/2 pulse width of 6.25 μs and a recycle delay (d1) of 30 s.

ssNMR analysis of the water-treated InP was performed at the Reactor Institute Delft. Samples dried in vacuo were mixed with activated alumina and loaded into a 4 mm zirconia rotor. Measurements were then performed with a Bruker Acsend 500 magnet (11.7 T) with a NEO console operating at a ^31^P resonance frequency of 202.45 MHz and a MAS spinning frequency of 8 kHz. Spectra were referenced to external H_3_PO_4_ (=0 ppm). Single pulse measurements were performed with a recycle delay (d_1_) of 50 s and a pulse width of 4.8 μs. Proton decoupling was performed during the measurement using the Spinal-64 decoupling sequence.

### XPS

Samples were prepared by drop casting the QD dispersions gold-plated glass substrates inside a nitrogen-filled glovebox and were vacuum-transferred to the instrument to avoid exposure to air. Measurements were performed under UHV (<2 × 10^–7^ mbar) on a ThermoFisher K-Alpha equipped with Al Kα source, radiating with an energy of 1486 eV. A flood gun (Ar) was active during all measurements to prevent charging of the samples. Samples were etched with an ion gun before measurement for 30 s to avoid surface bias and measure a representative average of the QD sample.

### DFT Calculations

Crystal structures were obtained from the Materials Project,^28^ specifics are available in Table S1. Before calculating the chemical shieldings, all structures were optimized at the PBE level.^30,31^ An epitaxial ZnSe/InP interface, featuring 10 atomic layers of each compound, was constructed based on ZnSe lattice parameters obtained from the previous geometry optimization. The interface model includes no vacuum, and is essentially an infinite repetition of alternating ZnSe/InP layers. Consecutively, we relaxed the atomic positions while varying the long direction of the unit cell to find the minimum energy structure (Figure S9). All DFT calculations presented in this work were performed using VASP,^32,33^ using the projector augmented-wave (PAW)^34,35^ and gauge-including PAW methods.^36^ Detailed input parameters are available in the Supporting Information Section S1.

## Supporting Information Available

Additional absorbance, photoluminescence, HR-TEM, solution NMR, ssNMR and XPS data, details of DFT calculations (PDF)

## References

[ref1] Almeida G.; Ubbink R. F.; Stam M.; du Fossé I.; Houtepen A. J. InP colloidal quantum dots for visible and near-infrared photonics. Nat. Rev. Mater. 2023, 8 (11), 742–758. 10.1038/s41578-023-00596-4.

[ref2] Chen B.; Li D.; Wang F. InP quantum dots: synthesis and lighting applications. Small 2020, 16 (32), 2002454 10.1002/smll.202002454. 32613755

[ref3] Wu Z.; Liu P.; Zhang W.; Wang K.; Sun X. W. Development of InP quantum dot-based light-emitting diodes. ACS Energy Lett. 2020, 5 (4), 1095–1106. 10.1021/acsenergylett.9b02824.

[ref4] Won Y.-H.; Cho O.; Kim T.; Chung D.-Y.; Kim T.; Chung H.; Jang H.; Lee J.; Kim D.; Jang E. Highly efficient and stable InP/ZnSe/ZnS quantum dot light-emitting diodes. Nature 2019, 575 (7784), 634–638. 10.1038/s41586-019-1771-5. 31776489

[ref5] Van Avermaet H.; Schiettecatte P.; Hinz S.; Giordano L.; Ferrari F.; Nayral C.; Delpech F.; Maultzsch J.; Lange H.; Hens Z. Full-Spectrum InP-Based Quantum Dots with Near-Unity Photoluminescence Quantum Efficiency. ACS Nano 2022, 16 (6), 9701–9712. 10.1021/acsnano.2c03138. 35709384

[ref6] Jo D.-Y.; Kim H.-M.; Park G. M.; Shin D.; Kim Y.; Kim Y.-H.; Ryu C. W.; Yang H. Unity quantum yield of InP/ZnSe/ZnS quantum dots enabled by Zn halide-derived hybrid shelling approach. Soft Sci. 2024, 4 (3), 27 10.20517/ss.2024.19.

[ref7] Li Y.; Hou X.; Dai X.; Yao Z.; Lv L.; Jin Y.; Peng X. Stoichiometry-controlled InP-based quantum dots: synthesis, photoluminescence, and electroluminescence. J. Am. Chem. Soc. 2019, 141 (16), 6448–6452. 10.1021/jacs.8b12908. 30964282

[ref8] Kim Y.; Ham S.; Jang H.; Min J. H.; Chung H.; Lee J.; Kim D.; Jang E. Bright and uniform green light emitting InP/ZnSe/ZnS quantum dots for wide color gamut displays. ACS Appl. Nano Mater. 2019, 2 (3), 1496–1504. 10.1021/acsanm.8b02063.

[ref9] Cui Z.; Qin S.; He H.; Wen Z.; Yang D.; Piao Z.; Mei S.; Zhang W.; Guo R. Sequential Growth of InP Quantum Dots and Coordination between Interfacial Heterovalency and Shell Confinement: Implication for Light-Emitting Devices. ACS Appl. Nano Mater. 2023, 7, 1181–1190. 10.1021/acsanm.3c05167.

[ref10] Jeong B. G.; Chang J. H.; Hahm D.; Rhee S.; Park M.; Lee S.; Kim Y.; Shin D.; Park J. W.; Lee C.; Lee D. C.; Park K.; Hwang E.; Bae W. K. Interface polarization in heterovalent core-shell nanocrystals. Nat. Mater. 2021, 21, 246–252. 10.1038/s41563-021-01119-8. 34795403

[ref11] Tessier M. D.; Baquero E. A.; Dupont D.; Grigel V.; Bladt E.; Bals S.; Coppel Y.; Hens Z.; Nayral C.; Delpech F. Interfacial oxidation and photoluminescence of InP-based core/shell quantum dots. Chem. Mater. 2018, 30 (19), 6877–6883. 10.1021/acs.chemmater.8b03117.

[ref12] Vikram A.; Zahid A.; Bhargava S. S.; Jang H.; Sutrisno A.; Khare A.; Trefonas P.; Shim M.; Kenis P. J. Unraveling the origin of interfacial oxidation of InP-based quantum dots: implications for bioimaging and optoelectronics. ACS Appl. Nano Mater. 2020, 3 (12), 12325–12333. 10.1021/acsanm.0c02814.

[ref13] Choi Y.; Hahm D.; Bae W. K.; Lim J. Heteroepitaxial chemistry of zinc chalcogenides on InP nanocrystals for defect-free interfaces with atomic uniformity. Nat. Commun. 2023, 14 (1), 43 10.1038/s41467-022-35731-2. 36596807 PMC9810615

[ref14] Stam M.; Almeida G.; Ubbink R. F.; van der Poll L. M.; Vogel Y. B.; Chen H.; Giordano L.; Schiettecatte P.; Hens Z.; Houtepen A. J. Near-Unity Photoluminescence Quantum Yield of Core-Only InP Quantum Dots via a Simple Postsynthetic InF3 Treatment. ACS Nano 2024, 18, 14685–14695. 10.1021/acsnano.4c03290. 38773944 PMC11155241

[ref15] Dumbgen K. C.; Leemans J.; De Roo V.; Minjauw M.; Detavernier C.; Hens Z. Surface Chemistry of InP Quantum Dots, Amine-Halide Co-Passivation, and Binding of Z-Type Ligands. Chem. Mater. 2023, 35 (3), 1037–1046. 10.1021/acs.chemmater.2c02960.

[ref16] Tomaselli M.; Yarger J.; Bruchez M.; Havlin R.; DeGraw D.; Pines A.; Alivisatos A. NMR study of InP quantum dots: Surface structure and size effects. J. Chem. Phys. 1999, 110 (18), 8861–8864. 10.1063/1.478858.

[ref17] Cros-Gagneux A.; Delpech F.; Nayral C.; Cornejo A.; Coppel Y.; Chaudret B. Surface chemistry of InP quantum dots: a comprehensive study. J. Am. Chem. Soc. 2010, 132 (51), 18147–18157. 10.1021/ja104673y. 21126088

[ref18] Virieux H.; Le Troedec M.; Cros-Gagneux A.; Ojo W.-S.; Delpech F.; Nayral C.; Martinez H.; Chaudret B. InP/ZnS nanocrystals: coupling NMR and XPS for fine surface and interface description. J. Am. Chem. Soc. 2012, 134 (48), 19701–19708. 10.1021/ja307124m. 23131073

[ref19] Ubbink R. F.; Almeida G.; Iziyi H.; Du Fossé I.; Verkleij R.; Ganapathy S.; Van Eck E. R.; Houtepen A. J. A Water-Free In Situ HF Treatment for Ultrabright InP Quantum Dots. Chem. Mater. 2022, 34 (22), 10093–10103. 10.1021/acs.chemmater.2c02800. 36439318 PMC9686131

[ref20] Wang F.; Tang R.; Buhro W. E. The trouble with TOPO; identification of adventitious impurities beneficial to the growth of cadmium selenide quantum dots, rods, and wires. Nano Lett. 2008, 8 (10), 3521–3524. 10.1021/nl801692g. 18754691

[ref21] Kolodziejski W.; Klinowski J. Kinetics of cross-polarization in solid-state NMR: a guide for chemists. Chem. Rev. 2002, 102 (3), 613–628. 10.1021/cr000060n. 11890752

[ref22] Cadars S.; Smith B.; Epping J.; Acharya S.; Belman N.; Golan Y.; Chmelka B. Atomic positional versus electronic order in semiconducting ZnSe nanoparticles. Phys. Rev. Lett. 2009, 103 (13), 136802 10.1103/PhysRevLett.103.136802. 19905534

[ref23] Demko B. A.; Wasylishen R. E. Solid-state selenium-77 NMR. Prog. Nucl. Magn. Reson. Spectrosc. 2009, 54 (3–4), 208–238. 10.1016/j.pnmrs.2008.10.002.

[ref24] Zhu D.; Bellato F.; Bahmani Jalali H.; Di Stasio F.; Prato M.; Ivanov Y. P.; Divitini G.; Infante I.; De Trizio L.; Manna L. ZnCl2Mediated synthesis of InAs nanocrystals with aminoarsine. J. Am. Chem. Soc. 2022, 144 (23), 10515–10523. 10.1021/jacs.2c02994. 35648676 PMC9204758

[ref25] Zhu D.; Bahmani Jalali H.; Saleh G.; Di Stasio F.; Prato M.; Polykarpou N.; Othonos A.; Christodoulou S.; Ivanov Y. P.; Divitini G.; et al. Boosting the Photoluminescence Efficiency of InAs Nanocrystals Synthesized with Aminoarsine via a ZnSe Thick-Shell Overgrowth. Adv. Mater. 2023, 35 (38), 2303621 10.1002/adma.202303621. 37243572

[ref26] Han Q.; Gao P.; Liang L.; Chen K.; Dong A.; Liu Z.; Han X.; Fu Q.; Hou G. Unraveling the surface hydroxyl network on In2O3 nanoparticles with high-field ultrafast magic angle spinning nuclear magnetic resonance spectroscopy. Anal. Chem. 2021, 93 (50), 16769–16778. 10.1021/acs.analchem.1c02759. 34878248

[ref27] Almeida G.; van der Poll L.; Evers W. H.; Szoboszlai E.; Vonk S. J.; Rabouw F. T.; Houtepen A. J. Size-dependent optical properties of InP colloidal quantum dots. Nano Lett. 2023, 23 (18), 8697–8703. 10.1021/acs.nanolett.3c02630. 37672486 PMC10540257

[ref28] Jain A.; Ong S. P.; Hautier G.; Chen W.; Richards W. D.; Dacek S.; Cholia S.; Gunter D.; Skinner D.; Ceder G.; Persson K. A. Commentary: The Materials Project: A materials genome approach to accelerating materials innovation. APL Mater. 2013, 1 (1), 011002 10.1063/1.4812323.

[ref29] Brouwer A. M. Standards for photoluminescence quantum yield measurements in solution (IUPAC Technical Report). Pure Appl. Chem. 2011, 83 (12), 2213–2228. 10.1351/PAC-REP-10-09-31.

[ref30] Perdew J. P.; Burke K.; Ernzerhof M. Generalized gradient approximation made simple. Phys. Rev. Lett. 1996, 77 (18), 3865 10.1103/PhysRevLett.77.3865. 10062328

[ref31] Perdew J. P.; Burke K.; Ernzerhof M. Generalized Gradient Approximation Made Simple [Phys. Rev. Lett. 77, 3865 (1996)]. Phys. Rev. Lett. 1997, 78 (7), 1396 10.1103/PhysRevLett.78.1396. 10062328

[ref32] Kresse G.; Furthmüller J. Efficient iterative schemes for ab initio total-energy calculations using a plane-wave basis set. Phys. Rev. B 1996, 54 (16), 11169–11186. 10.1103/PhysRevB.54.11169. 9984901

[ref33] Kresse G.; Furthmüller J. Efficiency of ab-initio total energy calculations for metals and semiconductors using a plane-wave basis set. Comput. Mater. Sci. 1996, 6 (1), 15–50. 10.1016/0927-0256(96)00008-0.

[ref34] Blöchl P. E. Projector augmented-wave method. Phys. Rev. B 1994, 50 (24), 17953 10.1103/PhysRevB.50.17953. 9976227

[ref35] Kresse G.; Joubert D. From ultrasoft pseudopotentials to the projector augmented-wave method. Phys. Rev. B 1999, 59 (3), 1758 10.1103/PhysRevB.59.1758.

[ref36] Yates J. R.; Pickard C. J.; Mauri F. Calculation of NMR chemical shifts for extended systems using ultrasoft pseudopotentials. Phys. Rev. B:Condens. Matter Mater. Phys. 2007, 76 (2), 024401 10.1103/physrevb.76.024401.

