# Supplementary material for: Phosphorus Oxidation Controls Epitaxial Shell Growth in InP/ZnSe Quantum Dots
Source: ACS Nano. 2024 Dec 30;19(1):1150–8. doi: 10.1021/acsnano.4c13110 (PMC11752496; doi:10.1021/acsnano.4c13110)
Supplement: Supplementary file 1 — nn4c13110_si_001.pdf [file nn4c13110_si_001.pdf]

Supporting information for

Phosphorus Oxidation Controls Epitaxial Shell Growth in InP/ZnSe Quantum Dots

*Reinout F. Ubbink†, Tom Speelman§, Daniel Arenas Esteban††, Mourijn van Leeuwen†, Maarten Stam†, Sara Bals††, Gilles A. de Wijs§, Ernst R. H. van Eck§ and Arjan J. Houtepen†**

† Optoelectronic Materials Section, Faculty of Applied Sciences, Delft University of Technology, Van der Maasweg 9, 2629 HZ Delft, The Netherlands

†† EMAT Electron Microscopy for Materials Science, Department of Physics, University of Antwerp, Antwerp 2020, Belgium

§ Radboud University, Institute for Molecules and Materials, Heyendaalseweg 135, 6525 AJ Nijmegen, The Netherlands

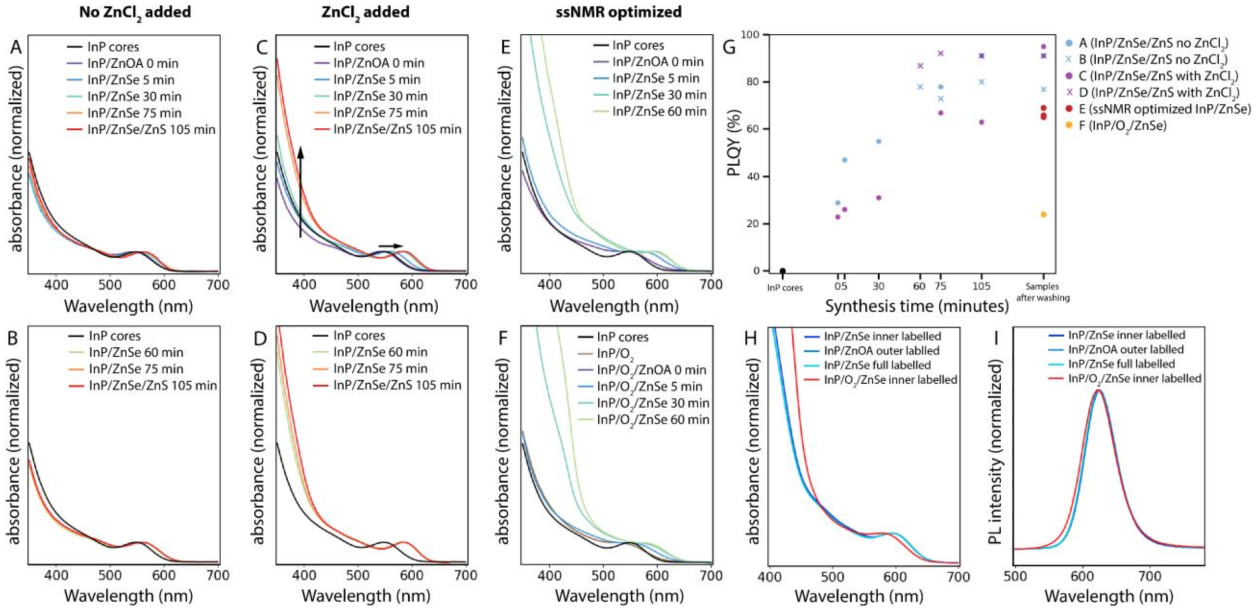

Figure S1. Overview of optical properties of the samples during synthesis and after purification. A), B) Absorption spectra of aliquots taken during shelling optimized for PLQY except no $ZnCl_2$ is added. C), D) Absorption spectra of aliquots taken during shelling optimized for PLQY where $ZnCl_2$ is added. Compared to shelling without $ZnCl_2$, a stronger redshift of the 1S peak and a larger increase at lower wavelengths is observed (as indicated by the arrows in C). This indicates the growth of a thicker shell when $ZnCl_2$ is added. Sample D) was measured using ssNMR (Figure S5). E) Absorption spectra of aliquots taken during the synthesis of the interface-^{77}Se labeled sample. By using TOA as the solvent (as opposed to ODE in the other samples), a thicker shell is grown, similar to the case where $ZnCl_2$ is added. The larger ZnSe shell allows more signal to be obtained from the samples in ssNMR. F) Absorption spectra of sample oxidized with molecular O_2 gas before the shelling procedure. The shelling procedure still works in the same way as for the non-oxidized samples. Compared to non-oxidized samples, the 1S peak of the final InP/ZnSe QDs is only slightly blueshifted and slightly broadened. G) PLQY values of all samples during the synthesis and after washing. The PLQY-optimized version of the protocol yields high-quality

InP/ZnSe/ZnS QDs with PLQY values >90%. ssNMR-optimized samples show lower PLQY values (around 66%), likely due to the missing ZnS outer shell in combination with the aggressive washing procedure used to prepare the samples for ssNMR analysis, which may increase surface defects.[1] The interface-oxidized sample shows significantly lower PLQY compared to ssNMR-optimized samples of 24%. H) Absorption spectra and I) photoluminescence spectra of all samples analyzed by ssNMR. All unoxidized InP/ZnSe particles synthesized have optical properties that are practically identical, regardless of the use of enriched ^{77}Se. Interface-oxidized InP/O_2/ZnSe particles show slightly broadened and redshifted emission and absorption compared to unoxidized ones. The increased intensity at lower wavelengths in the absorption spectrum of InP/O_2/ZnSe indicates that slightly thicker ZnSe shells are formed on that sample.

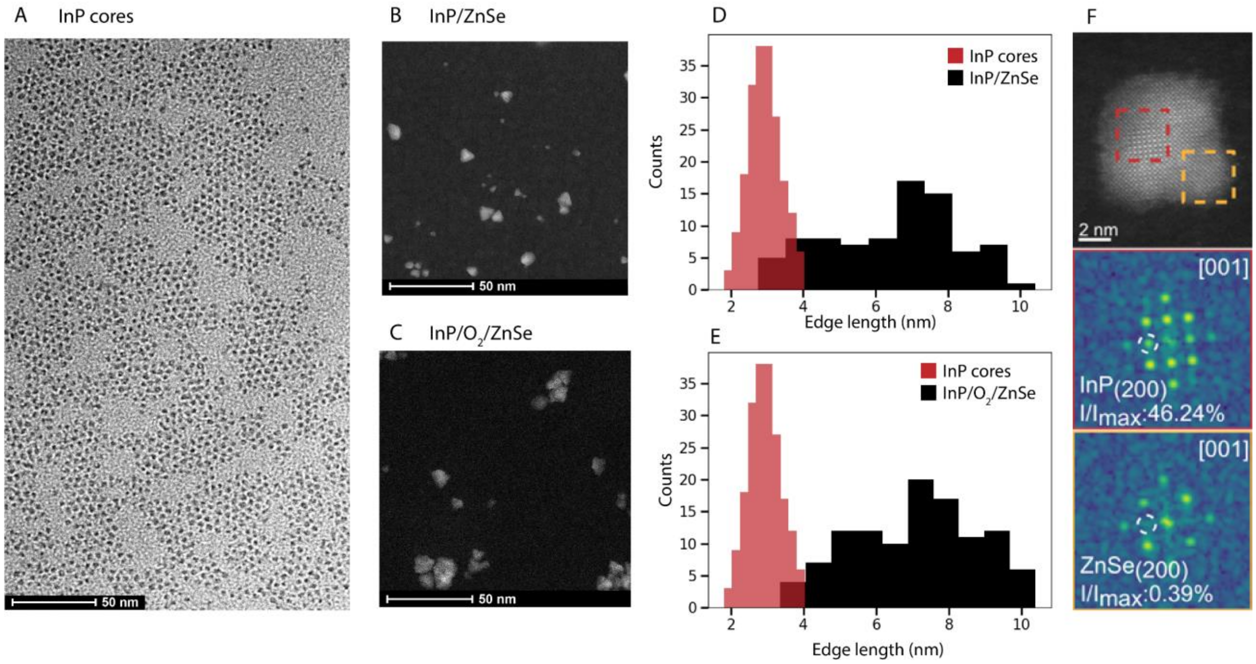

Figure S2. A) TEM images of InP core QDs. B), C) HAADF-STEM images of InP/ZnSe and InP/O_2/ZnSe QDs. D), E) Size histograms of InP core, InP/ZnSe and InP/O_2/ZnSe QDs edge lengths as measured from the TEM images. Mean edge lengths ± 2 standard deviations were calculated from this distribution. InP cores: 2.9 ± 0.90 nm, InP/ZnSe: 6.5 ± 3.6 nm, InP/O_2/ZnSe: 7.1 ± 3.4 nm, indicating a slightly thicker shell on the InP/O_2/ZnSe sample in correspondence with absorption and XRD measurements. F) HAADF-STEM image of one the unoxidized QDs oriented along the 001 zone axis. The bottom insets show the FFT analysis in the center (red) and a corner (orange) of the QD, where a characteristic reduction in the intensity of the (200) spacing, highlighted with a dashed white circle, can be observed from the InP to the ZnSe cubic crystal structures.

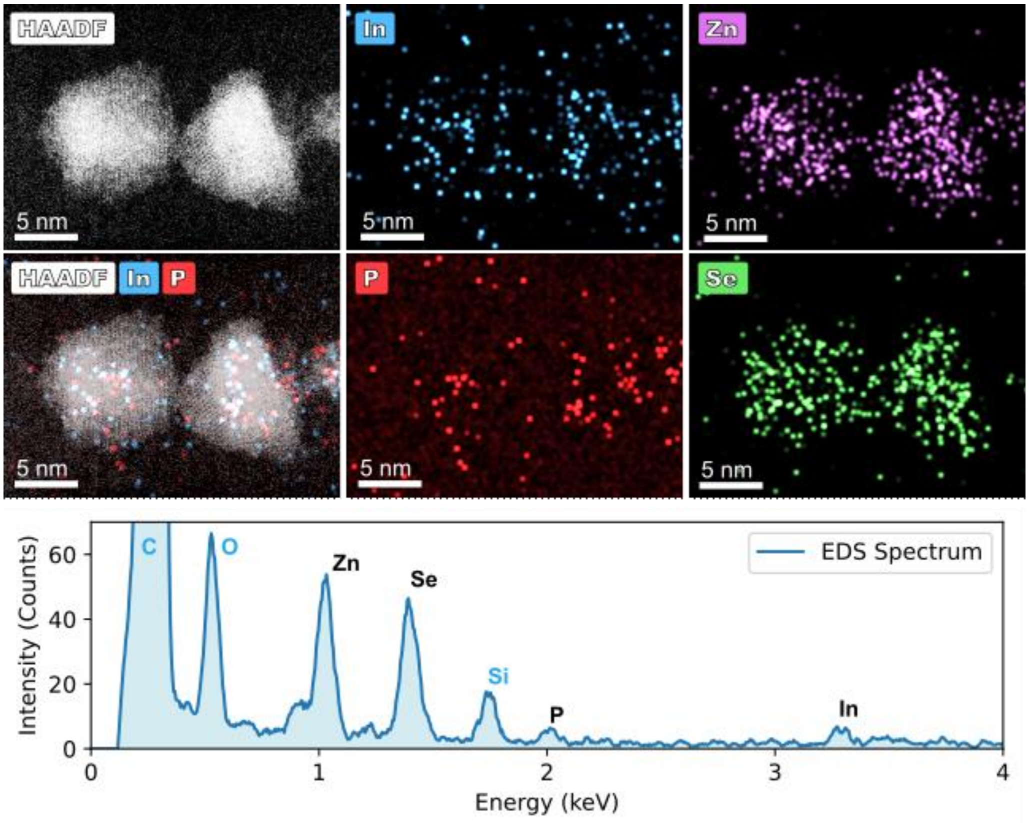

Figure S3. EDS analysis on 2 InP/ZnSe core-shell QDs. The elemental map distribution shows a concentration of In and P inside the nanoparticles as well as Zn and Se at the external parts. In, P, Zn, and Se peaks can be detected on the EDS spectrum together with C, O, and Si that can be ascribed to the specimen support.

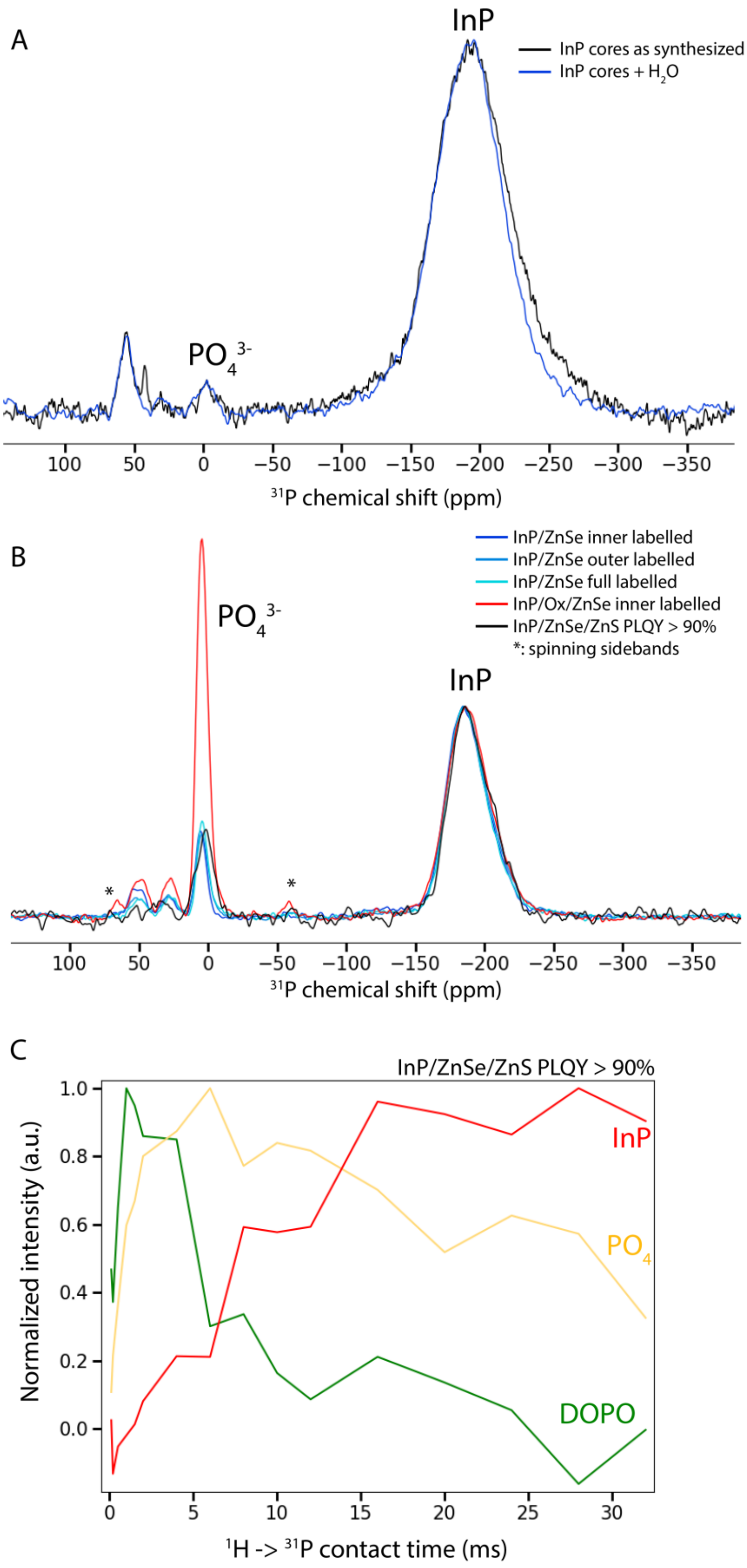

A
InP
InP cores as synthesized
InP cores + H_2O
PO_4^{3-}
100 50 0 −50 −100 −150 −200 −250 −300 −350
^{31}P chemical shift (ppm)
B
InP/ZnSe inner labelled
InP/ZnSe outer labelled
InP/ZnSe full labelled
InP/Ox/ZnSe inner labelled
InP/ZnSe/ZnS PLQY > 90%
*: spinning sidebands
PO_4^{3-}
InP
100 50 0 −50 −100 −150 −200 −250 −300 −350
^{31}P chemical shift (ppm)
C
InP/ZnSe/ZnS PLQY > 90%
Normalized intensity (a.u.)
1.0 0.8 0.6 0.4 0.2 0.0
InP
PO_4
DOPO
0 5 10 15 20 25 30
^1H -> ^{31}P contact time (ms)

Figure S4. A) ^{31}P ssNMR spectra of InP cores as synthesized and after attempted oxidation using water. Addition of water to the InP core solution at 120 °C did not result in an increase in PO_4^{3-} (peak around 0 ppm). B) ^{31}P ssNMR spectra of various InP/ZnSe and InP/ZnSe/ZnS samples. Regardless of the shelling method, the ratio of integrals of PO_4^{3-}/InP peaks is the same. Significant quantities of PO_4^{3-} are observed even on samples with PLQY > 90%. Only when the interface is oxidized on purpose using molecular O_2 is the amount of PO_4^{3-} significantly different. C) Intensity of DOPO (green), PO_4^{3-} (yellow) and InP (red) resonance in the InP/ZnSe/ZnS sample for different ^{1}H->^{31}P cross-polarization contact times. High intensities at short contact times indicate proximity to the hydrogen-rich ligands on the outer surface, while longer risetimes of the intensity indicate a position deeper inside the QD, removed from the surface ligands. The same trend in observed in the InP/ZnSe/ZnS sample as in the InP/ZnSe samples discussed in the main text in Figure 2B. DOPO intensity peaks at short contact times (~1 ms), confirming proximity to hydrogen and presence at the outer ZnS surface. The InP resonance intensity peaks at contact times >20 ms, because the core is far removed from the hydrogen-rich ligands. PO_4^{3-} intensity peaks at intermediate contact times (6 ms), indicating the position of PO_4^{3-} is at the interface, closer to the surface than InP, but still separated from the surface by the ZnSe and ZnS shells.

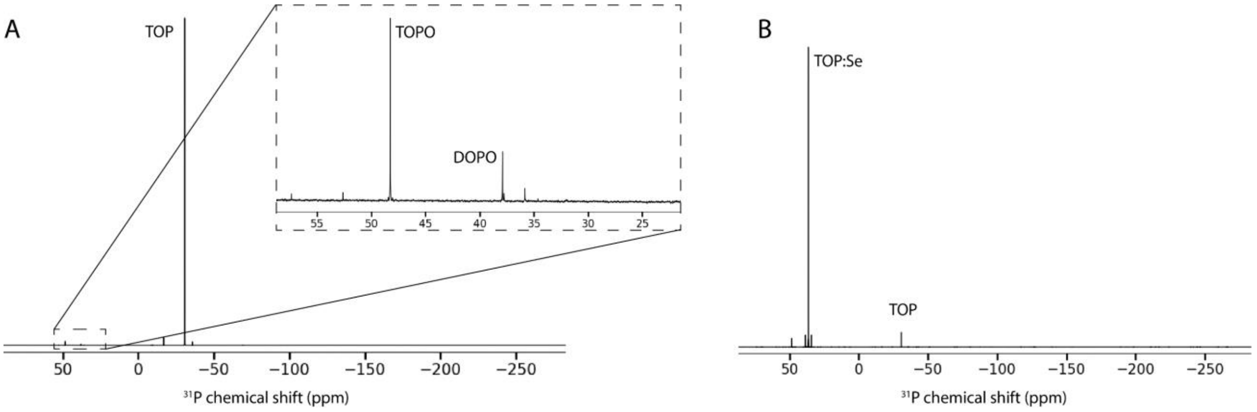

Figure S5. A) ^{1}H NMR of as-purchased TOP. TOPO and DOPO impurities can be observed.[2] B) ^{1}H NMR of elemental selenium dissolved in TOP. This compound is used as the Se precursor during shelling.

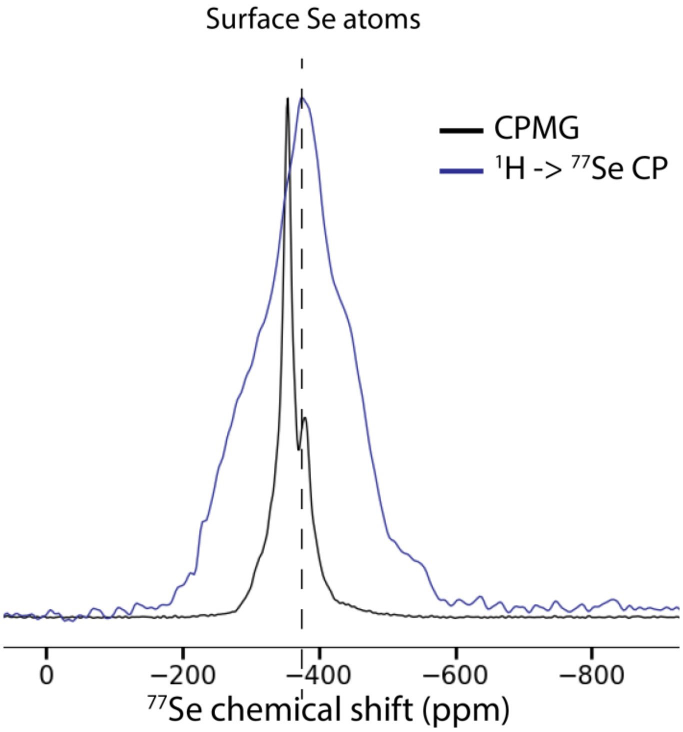

Figure S6. When ^{1}H->^{77}Se cross-polarization is employed, selenium signal of nuclei in close proximity to hydrogen (present only in surface ligands) is enhanced. It is observed that the signal around 380 ppm is enhanced, which is thus ascribed to selenium atoms at the outer ZnSe surface close the hydrogen-rich ligands. This is in agreement with REDOR measurements.

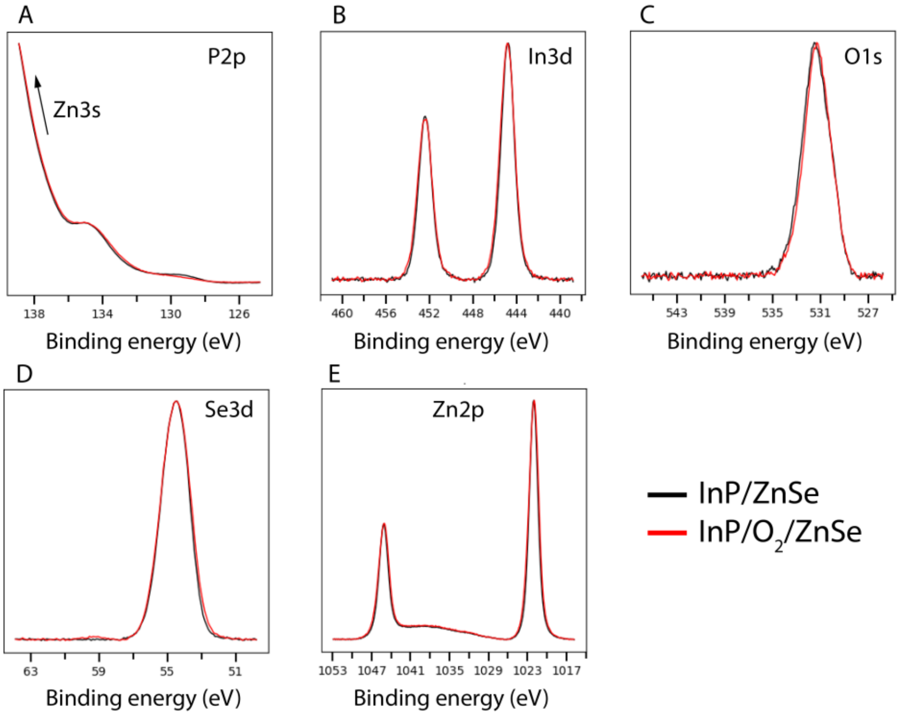

Figure S7. No difference is observed between the InP/ZnSe and InP/O_2/ZnSe in XPS data.

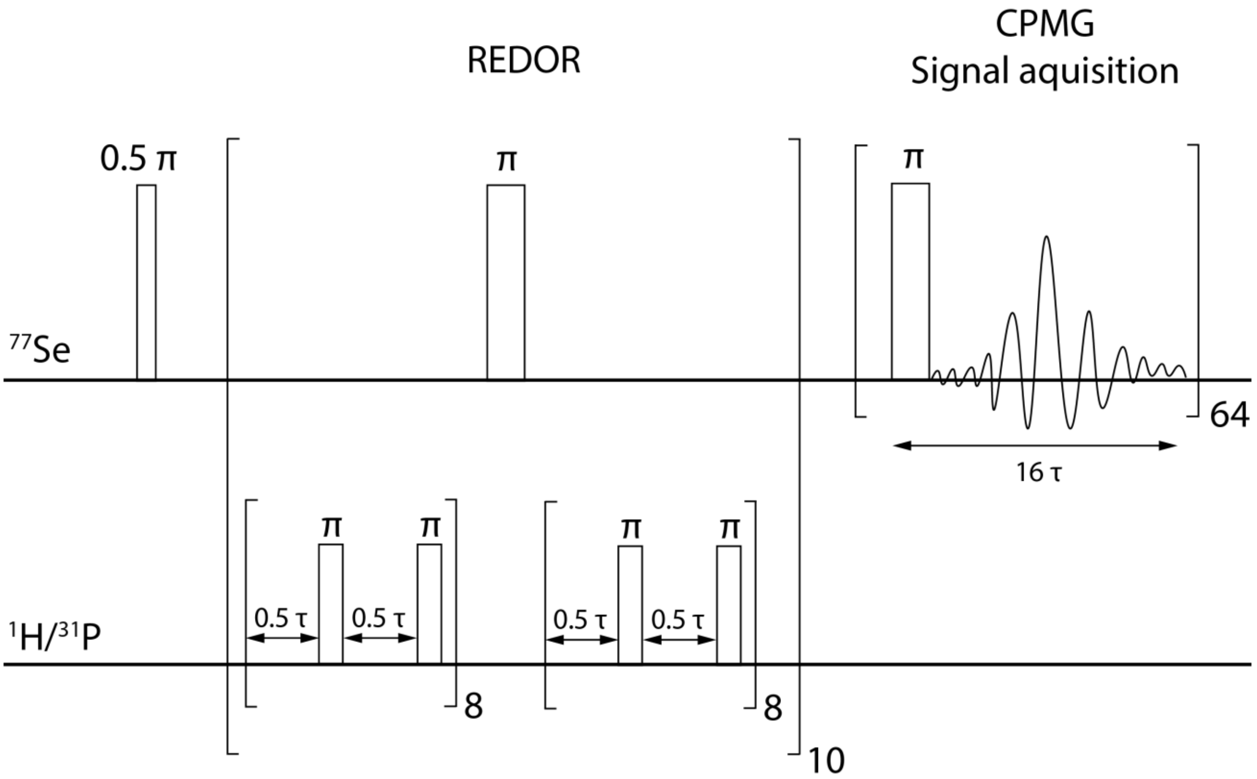

Figure S8. Pulse program employed to measure ^{77}Se in the InP/ZnSe QDs. All ^{77}Se measurements were performed using the CPMG detection sequence, employing 64 detection cycles with a 180° refocusing pulse in between. For the REDOR interaction measurements, the REDOR dephasing sequence was performed for 16 ms (160 rotor periods) before the signal acquisition was started. A long recycle delay (d1) of 300 s was always used in between scans.

Section S1: DFT calculations

This section describes the DFT calculations performed. First the bulk structures will be discussed, followed by the ZnSeInP interface model.

Calculations report chemical shieldings (σ), we relate these to experiment such that the bulk ZnSe chemical shifts (δ) coincide, i.e. $\delta_{calc} = \delta_{exp}$. We use the following formula:

$$\delta_{calc} = \delta_{exp,\,ZnSe} - \sigma_{calc} + \sigma_{calc,\,ZnSe}.$$

For the bulk structures $\sigma_{calc,\,ZnSe}$ is obtained from an explicit calculation on ZnSe. However, for the interface we select the ^{77}Se shielding from the center of the ZnSe layer as $\sigma_{calc,\,ZnSe}$ instead.

Section S1.1: Bulk Structures

In addition to the k-point meshes specified below, for the geometry optimizations we used a cut-off energy of 520 eV with an electronic convergence criterion of 1E-07 eV (EDIFF) and ionic convergence of -1E-03 eV (EDIFFG). Pseudopotentials used were the VASP "Zn", "In_d", and "Se" PBE.54 POTCARs. Materials Project IDs of input structures and k-point meshes are listed below (table S1).

Chemical shieldings were calculated with a cut-off energy of 520 eV and electronic convergence criterion of 1E-10 eV, employing the same pseudopotentials as before.

Table S1. Input structures for geometry optimization and k-point meshes of geometry optimization and chemical shielding calculation for bulk structures.

Compound (Materials Project ID)	**Space Group**	**k**-point mesh (gamma-centered)	
		Geometry Optimization	Shielding Calculation
In_2Se_3 (MP-612740)	$P6_1$	7×7×3	15×15×5
$ZnIn_2Se_4$ (MP-22607)	$I\bar{4}$	8×8×4	20×20×10
ZnSe (MP-1190)	$F\bar{4}3m$	8×8×8	32×32×32

Section S1.1.1: Relaxed Geometries (VASP POSCAR format)

In_2Se_3

```
In12 Se18
  1.00000000000000
    3.6756387541508158  -6.3663983145751306   0.0000000000000000
    3.6756432939570898   6.3663956935167576   0.0000000000000000
    0.0000000000000000   0.0000000000000000  19.9285931157054783
  In   Se
   12    18
Direct
  0.3383031550647440  0.9916157046693755  0.0010428330381955
  0.0083842953306244  0.3466874503953754  0.3343761663715313
 -0.0012812085365374  0.6946327748571240  0.4718358103379248
  0.0012812085365371  0.3053672251428763  0.9718358103379250
  0.9916157046693755  0.6533125496046246  0.8343761663715311
  0.3466874503953754  0.3383031550647440  0.1677094997048597
  0.6533125496046246  0.6616968449352562  0.6677094997048597
  0.6616968449352562  0.0083842953306244  0.5010428330381954
  0.3040860166063385 -0.0012812085365374  0.6385024770045893
  0.3053672251428763  0.3040860166063385  0.8051691436712607
  0.6959139833936612  0.0012812085365371  0.1385024770045890
  0.6946327748571240  0.6959139833936612  0.3051691436712605
  0.6833411579224427  0.0246539976982884  0.6349030355696801
  0.6296525901223928  0.6668745609784090  0.4513197735514310
  0.9365034060985102  0.2732680492009271  0.8288145375027723
  0.9627780291439847  0.6296525901223928  0.6179864402180949
  0.3413128397758383  0.3166588420775570  0.3015697022363511
  0.0634965939014899  0.7267319507990722  0.3288145375027723
  0.9753460023017116  0.6586871602241614  0.9682363689030158
  0.6668745609784090  0.0372219708560159  0.2846531068847596
  0.3166588420775570  0.9753460023017116  0.1349030355696797
  0.2732680492009271  0.3367646431024174  0.6621478708361009
  0.6632353568975831  0.9365034060985102  0.9954812041694366
  0.3367646431024173  0.0634965939014899  0.4954812041694366
  0.6586871602241614  0.6833411579224427  0.8015697022363515
  0.3331254390215912  0.9627780291439847  0.7846531068847592
  0.7267319507990722  0.6632353568975831  0.1621478708361011
  0.3703474098776068  0.3331254390215912  0.9513197735514306
  0.0372219708560159  0.3703474098776068  0.1179864402180951
  0.0246539976982884  0.3413128397758383  0.4682363689030154
```

Zn(InSe2)2

```
Zn2 In4 Se8
   1.00000000000000
     5.8152197002519976    0.0000003996473628    0.0000000000000004
    -0.0000003996473619    5.8152197002519976    0.0000000000000004
     0.0000000000000000    0.0000000000000000   11.7806870449870278
   Zn   In   Se
     2     4     8
Direct
 -0.0000000000000000  0.0000000000000000  0.0000000000000000
  0.5000000000000000  0.5000000000000000  0.5000000000000000
  0.5000000000000000  0.5000000000000000  0.0000000000000000
  0.5000000000000000  0.0000000000000000  0.2500000000000000
 -0.0000000000000000  0.0000000000000000  0.5000000000000000
 -0.0000000000000000  0.5000000000000000  0.7500000000000000
  0.7608158997820359  0.2813397586496779  0.3698900020945177
  0.2391841002179643  0.7186602413503220  0.3698900020945177
  0.2186602413503222  0.2608158997820358  0.1301099979054825
  0.7813397586496780  0.7391841002179641  0.1301099979054825
  0.2608158997820358  0.7813397586496780  0.8698900020945175
  0.7391841002179641  0.2186602413503222  0.8698900020945175
  0.7186602413503220  0.7608158997820359  0.6301099979054825
  0.2813397586496779  0.2391841002179643  0.6301099979054825
```

ZnSe

```
Zn4 Se4
   1.00000000000000
     5.7385390074228200    0.0000000000000000    0.0000000000000003
     0.0000000000000009    5.7385390074228200    0.0000000000000003
     0.0000000000000000    0.0000000000000000    5.7385390074228200
   Zn   Se
     4     4
Direct
 -0.0000000000000000 -0.0000000000000000 -0.0000000000000000
 -0.0000000000000000  0.5000000000000000  0.5000000000000000
  0.5000000000000000  0.0000000000000000  0.5000000000000000
  0.5000000000000000  0.5000000000000000 -0.0000000000000000
  0.7500000000000000  0.2500000000000000  0.2500000000000000
  0.7500000000000000  0.7500000000000000  0.7500000000000000
  0.2500000000000000  0.2500000000000000  0.7500000000000000
  0.2500000000000000  0.7500000000000000  0.2500000000000000
```

Section S1.1.2: ^{77}Se Chemical Shielding Tensors (including susceptibility)

We present the shielding tensors with the applied field direction along the columns and the induced field along the rows. For each tensor, the corresponding atom number in the compound's POSCAR is mentioned along with its Wyckoff position.

In_2Se_3

Atom 13, Wyckoff 6a: x=0.68334, y=0.02465, z=0.63490

σ (ppm)	x	y	z
x	784.2	-716.0	-35.5
y	-357.0	1149.3	83.7
z	-164.7	-39.4	1315.6

Atom 14, Wyckoff 6a: x=0.62965, y=0.66687, z=0.45132

σ (ppm)	x	y	z
x	803.4	-576.7	-344.9
y	-436.6	781.3	-243.6
z	19.7	-87.4	1315.6

Atom 15, Wyckoff 6a: x=0.93650, y=0.27327, z=0.82881

σ (ppm)	x	y	z
x	300.4	-119.2	-40.4
y	-286.4	475.8	-5.7
z	3.5	8.1	1344.2

$ZnIn_2Se_4$

Atom 7, Wyckoff 8g: x=0.76082, y=0.28134, z=0.36989

σ (ppm)	x	y	z
x	1457.3	45.3	120.6
y	53.1	1473.3	-5.0
z	-4.9	-115.6	1413.1

ZnSe

Atom 5, Wyckoff 4d: x=0.75000, y=0.25000, z=0.25000

σ (ppm)	x	y	z
x	1789.1	0.0	0.0
y	0.0	1789.1	0.0
z	0.0	0.0	1789.1

Section S1.2: Interface

Starting from the optimized cubic ZnSe structure, an epitaxial [110] interface was constructed of 10 layers ZnSe and 10 layers of InP using the ZnSe lattice parameters. Fixing the **b** and **c**-lattice vectors, we varied the length of the **a**-vector to find the optimal value. We used a cut-off energy of 520 eV with an electronic convergence criterion of 1E-07 eV (EDIFF) and ionic convergence of -1E-03 eV (EDIFFG). Pseudopotentials used were the VASP "Zn", "In_d", and "Se" PBE.54 POTCARs. A 1×10×10 Gamma-centered **k**-point mesh was used.

For the optimal structure, chemical shieldings were calculated with a cut-off energy of 520 eV and electronic convergence of 1E-10 eV, employing the same pseudopotentials as before. The **k**-point sampling was increased to 2×16×16.

The isotropic shieldings reported in the main text are obtained using averages of the *xx*, *yy* and *zz* components of the shielding tensors. In spite of the local cubic symmetry at the Se nuclei far from the interfaces their shielding tensors are not isotropic due to the presence of induced net interface currents at the ZnSe/InP interfaces.

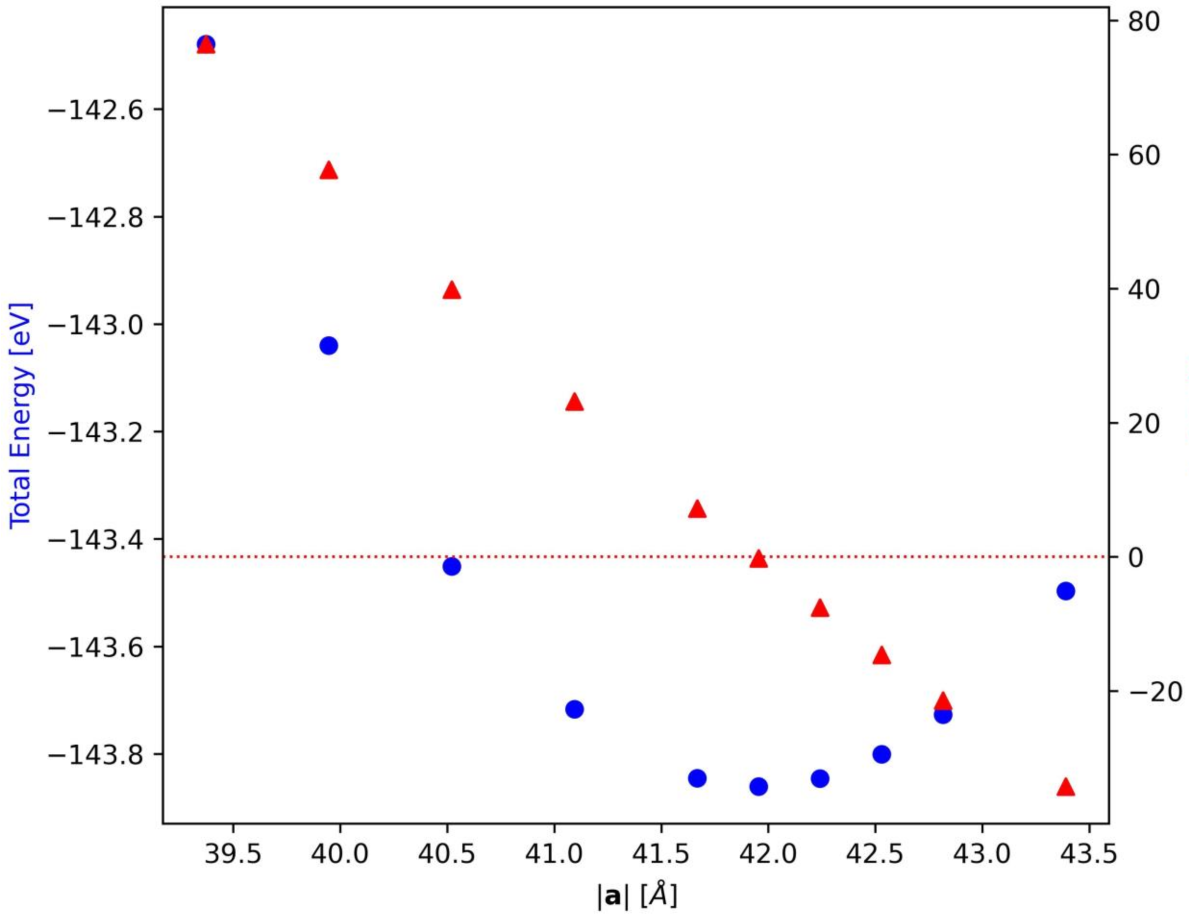

Figure S9. Total Energy [eV] (bullets) and *xx* component of the stress tensor P_x [kB] (triangles) versus **a**-vector length [Å] for the ZnSeInP-interface model.

Section S1.2.1: Relaxed geometry (VASP POSCAR format)

```
ZnSeInP
  5.73857036341909
    7.3110678118654757    0.0000000000000000    0.0000000000000000
    0.0000000000000000    0.7071067811865476    0.0000000000000000
    0.0000000000000000    0.0000000000000000    1.0000000000000000
  Zn   Se   In   P
   10    10    10    10
Direct
  0.0054957816046931  0.0000000000000000  0.0095509870268054
  0.0560867858712685  0.5000000000000000  0.4966324659027256
  0.1038581910075525  0.0000000000000000 -0.0008197355742266
  0.1524723592633789  0.5000000000000000  0.4990076603455083
  0.2007496674545740 -0.0000000000000000 -0.0008202940229541
  0.2492503325454288  0.5000000000000000  0.4991797059770388
  0.2975276407366167 -0.0000000000000000 -0.0009923396544918
  0.3461418089924502  0.5000000000000000  0.4991802644257736
  0.3939132141287273 -0.0000000000000000  0.9966324659027251
  0.4445042183953097  0.5000000000000000  0.5095509870268056
  0.0083664350605617  0.5000000000000000  0.7561268595721579
  0.0554069476111064 -0.0000000000000000  0.2484023300581214
  0.1040922545293080  0.5000000000000000  0.7493261852909381
  0.1523313460108733  0.0000000000000000  0.2489770608243115
  0.2008347253619538  0.5000000000000000  0.7491009405032172
  0.2491652746380491 -0.0000000000000000  0.2491009405032309
  0.2976686539891226  0.5000000000000000  0.7489770608243118
  0.3459077454707021 -0.0000000000000000  0.2493261852909450
  0.3945930523888894  0.5000000000000000  0.7484023300581208
  0.4416335649394411 -0.0000000000000000  0.2561268595721578
  0.4946723632300498  0.0000000000000000  0.9929240369683331
  0.5437860937982137  0.5000000000000000  0.5090858884984003
  0.5961141807491198 -0.0000000000000000  0.0078674392673148
  0.6473814204906000  0.5000000000000000  0.5087862984883545
  0.6991945459897825 -0.0000000000000000  0.0082238352535277
  0.7508054540102204  0.5000000000000000  0.5082238352535278
  0.8026185795093957 -0.0000000000000000  0.0087862984883475
  0.8538858192508830  0.5000000000000000  0.5078674392673148
  0.9062139062017820  0.0000000000000000  0.0090858884984001
  0.9553276367699530  0.5000000000000000  0.4929240369683329
  0.4912821013981162  0.5000000000000000  0.7345830571875491
  0.5446032989301618 -0.0000000000000000  0.2458899726796854
  0.5956819377143168  0.5000000000000000  0.7451734043763056
  0.6476147041011175 -0.0000000000000000  0.2462354646708546
  0.6991145808563176  0.5000000000000000  0.7457461426830431
  0.7508854191436852 -0.0000000000000000  0.2457461426830431
```

0.8023852958988782 0.5000000000000000 0.7462354646708544
0.8543180622856861 0.0000000000000000 0.2451734043763060
0.9053967010698339 0.5000000000000000 0.7458899726796855
0.9587178986018865 -0.0000000000000000 0.2345830571875488

Section S1.2.2: ^{77}Se Chemical Shielding Tensors (excluding susceptibility)

Due to the net induced interface currents, we report the chemical shielding tensors below without susceptibility (i.e. **G**=0 component). We list only the first 5 Se atoms from the POSCAR.

Atom 11

σ (ppm)	x	y	z
x	1717.8	0.0	16.6
y	0.0	1625.2	0.0
z	38.6	0.0	1701.5

Atom 12

σ (ppm)	x	y	z
x	1679.9	0.0	18.5
y	0.0	1713.0	0.0
z	1.8	0.0	1681.5

Atom 13

σ (ppm)	x	y	z
x	1749.8	0.0	2.4
y	0.0	1704.5	0.0
z	6.6	0.0	1716.1

Atom 14

σ (ppm)	x	y	z
x	1754.4	0.0	4.8
y	0.0	1750.9	0.0
z	1.7	0.0	1745.4

Atom 15

σ (ppm)	x	y	z
x	1760.2	0.0	-6.0
y	0.0	1758.4	0.0
z	0.0	0.0	1752.6

1. Schiettecatte, P.; Giordano, L.; Cruyssaert, B.; Bonifas, G.; De Vlamynck, N.; Van Avermaet, H.; Zhao, Q.; Vantomme, A.; Nayral, C.; Delpech, F., Enhanced Surface Passivation of InP/ZnSe Quantum Dots by Zinc Acetate Exposure. *Chemistry of Materials* **2024**.
2. Wang, F.; Tang, R.; Buhro, W. E., The trouble with TOPO; identification of adventitious impurities beneficial to the growth of cadmium selenide quantum dots, rods, and wires. *Nano letters* **2008,** *8* (10), 3521-3524.
